# A Natural History Study of Timothy Syndrome

**DOI:** 10.1186/s13023-024-03445-x

**Published:** 2024-11-23

**Authors:** Katherine W. Timothy, Rosemary Bauer, Kerry A. Larkin, Edward P. Walsh, Dominic J. Abrams, Cecilia Gonzalez Corcia, Alexandra Valsamakis, Geoffrey S. Pitt, Ivy E. Dick, Andy Golden

**Affiliations:** 1 The Timothy Syndrome Foundation, Charitable Organization, Brigham City, UT USA; 2 Laboratory of Biochemistry and Genetics, National Institute of Diabetes, Digestive, and Kidney Diseases, National Institute of Health, Bethesda, MD USA; 3 Division of Endocrinology, Metabolism, and Molecular Medicine, Department of Medicine, Northwestern University Feinberg School of Medicine, Chicago, IL USA; 4 Department of Cell Biology, Yale School of Medicine, 295 Congress Ave, New Haven, CT USA; 5 Department of Cardiology, Harvard Medical School, Boston Children’s Hospital, Boston, MA USA; 6 Department of Cardiology, Sainte Justine Hospital, Montreal, QC Canada; 7 Clinical Development and Medical Affairs, Roche Diagnostics Solutions, Pleasanton, CA USA; 8 Cardiovascular Research Institute, Weill Cornell Medicine, New York, NY USA; 9 Department of Physiology, School of Medicine, University of Maryland, Baltimore, MD USA

**Keywords:** Timothy syndrome, Long QT, Syndactyly, Hypoglycemia, Neurodevelopmental delay, *CACNA1C* mutation

## Abstract

**Background:**

Timothy syndrome (OMIM #601005) is a rare disease caused by variants in the gene *CACNA1C*. Initially, Timothy syndrome was characterized by a cardiac presentation of long QT syndrome and syndactyly of the fingers and/or toes, all associated with the *CACNA1C* variant, Gly406Arg. However, subsequent identification of diverse variants in *CACNA1C* has expanded the clinical spectrum, revealing various cardiac and extra-cardiac manifestations. It remains underexplored whether individuals with the canonical Gly406Arg variants in mutually exclusive exon 8A (Timothy syndrome 1) or exon 8 (Timothy syndrome 2) exhibit overlapping symptoms. Moreover, case reports have indicated that some *CACNA1C* variants may produce a cardiac-selective form of Timothy syndrome often referred to as non-syndromic long QT type 8 or cardiac-only Timothy syndrome, however few reports follow up on these patients to confirm the cardiac selectivity of the phenotype over time.

**Methods:**

A survey was administered to the parents of patients with Timothy syndrome, querying a broad range of symptoms and clinical features. Study participants were organized into 5 separate categories based on genotype and initial diagnosis, enabling comparison between groups of patients which have been described differentially in the literature.

**Results:**

Our findings reveal that Timothy syndrome patients commonly exhibit both cardiac and extra-cardiac features, with long QT syndrome, neurodevelopmental impairments, hypoglycemia, and respiratory issues being frequently reported. Notably, the incidence of these features was similar across all patient categories, including those diagnosed with non-syndromic long QT type 8, suggesting that the ‘non-syndromic’ classification may be incomplete.

**Conclusions:**

This study represents the first Natural History Study of Timothy syndrome, offering a comprehensive overview of the disease’s clinical manifestations. We demonstrate that both cardiac and extra-cardiac features are prevalent across all patient groups, underscoring the syndromic nature of *CACNA1C* variants. While the critical role of long QT syndrome and cardiac arrhythmias in Timothy syndrome has been well recognized, our findings indicate that hypoglycemia and respiratory dysfunction also pose significant life-threatening risks, emphasizing the need for comprehensive therapeutic management of affected individuals.

**Supplementary Information:**

The online version contains supplementary material available at 10.1186/s13023-024-03445-x.

## Background

In the early 1990s, after a flurry of publications identifying the genes responsible for many long QT (LQT) syndromes [1–5], several reports were published in which children were identified as having a prolonged QT interval and syndactyly [6–9]. At the time, Katherine Wilson Timothy (KWT) was the Clinical Coordinator for Dr. Mark T. Keating’s genetic research laboratory at the University of Utah which was searching the genome for variants that caused a prolonged QT interval and other cardiac abnormalities associated with sudden unexplained death (SUD) in the young. Recognizing these studies, cardiologists referred children to the laboratory with a specific LQT + syndactyly phenotype. Data from these children were collected, as this was believed to be a new syndrome. With 17 such children identified, most from the United States, Italy, New Zealand and the United Kingdom, the identity of the shared culprit gene was finally discovered after more than ten years of searching: *CACNA1C*, encoding the α_1C_ subunit of the voltage-gated L-type calcium channel Ca_v_1.2, which represents the dominant voltage-gated calcium channel within the heart. Thirteen of the 17 individuals had the identical missense variant, Gly406Arg, but DNA was not analyzed for the remaining four of these phenotypically identical children. Most children acquired this variant *de novo* [10]. The cardiac phenotypes presented by this autosomal dominant syndrome were thought to be the result of failed inactivation of Ca_v_1.2. Given that it was the eighth LQT gene to be identified molecularly, the LQT in these individuals was given the name LQT8. The syndrome was named Timothy syndrome (TS) (OMIM #601005) in recognition of KWT’s work. While TS was first identified due to shared LQT and syndactyly phenotypes among patients, it has become clear that these individuals experience variable symptoms across multiple organ systems. Thus, a systematic investigation into what phenotypes occur in TS is critical for proper identification and treatment of the disease.

The *CACNA1C* gene has at least 47 major exons and numerous splice variant products [11, 12]. An important difference between known splice forms is their inclusion of either exon 8 or 8A. Exons 8 and 8A are identical in size, and individual messenger RNAs derived from the *CACNA1C* gene either incorporate sequences from either exon 8 or 8A, but never both. Thus, we refer to them as being mutually exclusive.

The 13 original TS children had the identical variant in *CACNA1C* exon 8A, resulting in a missense change Gly406Arg [10]. This was originally defined as the cause of TS, later designated as TS type 1 (TS1), and was the first reported disease variant in *CACNA1C*. A case of TS (but without syndactyly) occurring in the alternative exon 8 was subsequently identified, which was then designated as TS type 2 (TS2) [13]. This second variant resulted in the homologous missense change Gly406Arg.

The Gly406Arg substitution has potent biophysical effects upon channel function, and these effects readily explain at least some of the phenotypes manifested by TS children. The LQT phenotype—the initial phenotype that brought these children to medical attention—is most readily explained. The Gly406Arg variant markedly decreases Ca_V_1.2 inactivation, leading to a markedly prolonged QT interval on the electrocardiogram [10, 13, 14], which is the substrate for the life-threatening arrhythmias. *CACNA1C*-encoded Ca_V_1.2 channels are broadly expressed including in non-excitable tissues [15], which provides a rationale for many of the phenotypes observed in TS children. For example, expression of Ca_V_1.2 channels in inhibitory neurons affects their migration during development and thus offers a possible explanation for the neurocognitive consequences of TS [16].

In the literature, the TS1 and TS2 types are often referred to as synonymous syndromes, mainly due to the identified similarities in their cardiac phenotypes. However, the development of many of the other systems (e.g., neuronal, bone, skeletal and smooth muscle development) are recognized to be differently affected. Exons 8 and 8A are used at distinct times and are dynamically regulated during mouse and human cerebral cortical development [17]. Similar regulation may occur in other organs or tissues. TS variants often arise de novo. The timing of occurrence can cause mosaicism leading to differential expression of mutant and wild type Ca_V_1.2 channels within specific tissues, complicating the comparison between TS1 and TS2 and among TS1 or TS2 affected individuals [18, 19].

An additional variant, Gly402Ser in exon 8, was identified in a separate individual concurrently with the TS2 change in 2005 [13]. It was reported in one individual who had LQT and neurological symptoms, but no syndactyly. Despite the different disease presentation from those with Gly406Arg, this case was classified as TS2 due to the phenotypic understanding at the time and the simultaneous discovery. In this study, Gly402Ser was analyzed separately from Gly406Arg TS1 and TS2 to enable comparison between the variants and the identification of unique phenotypes associated with a particular variant.

Numerous additional variants in the *CACNA1C* gene have been identified. Individuals with *CACNA1C* variants who have LQT but no immediately identified extra-cardiac symptoms typically received a diagnosis of LQT8. For the purpose of this study, we will separate these individuals and refer to them as diagnosed with non-syndromic LQT8 (nsLQT8). Individuals who have some symptom overlap with TS1 and TS2 have received a variety of diagnoses, including atypical TS, and *CACNA1C*-related disorder. While many of these individuals have been described in detail as individual case reports, we aim to analyze them together to highlight the many similarities in their disease presentation.

## Methods

### Inclusion

**Fig. 1:**
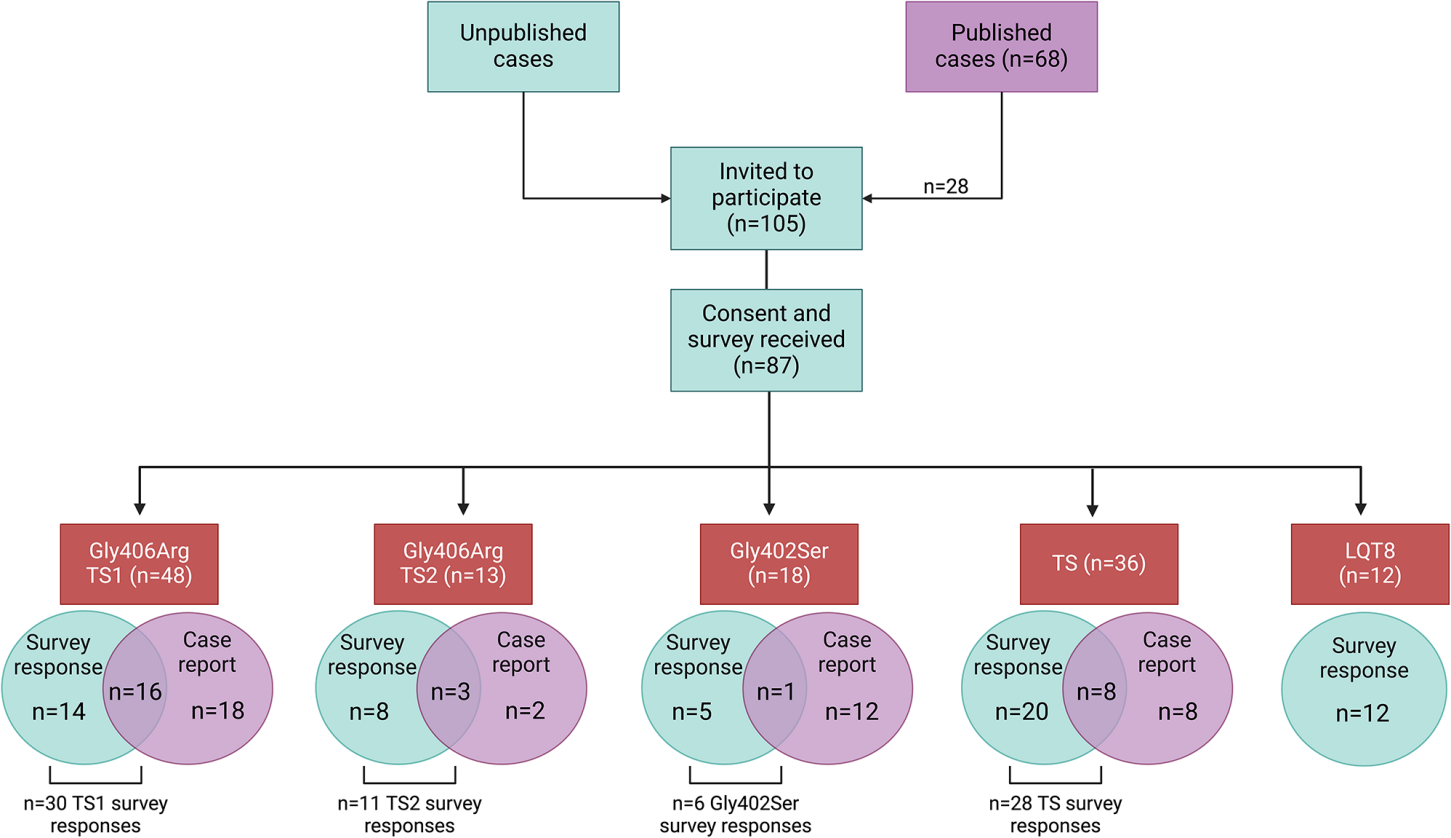
Schematic of the study participants. Blue indicates survey respondents, which comprise all data presented within the tables. Purple indicates cases published in the literature, with overlap of survey respondents as shown. Image created with BioRender.com

KWT has been the face of TS since it was first recognized as a syndrome and for the past three decades has served as the main point of contact for physicians and families when a child is first diagnosed. Continuous contact with many TS families has been maintained through in-person visits, attendance at annual Sudden Arrythmia Death Syndromes (SADS) and Timothy Syndrome Foundation Conference meetings, social media, email, phone calls, and letter writing. For this reason, we were able to invite 105 individuals to participate in this Natural History Study, having previously collected and retained their contact information (Fig. 1).

Study participation required the presence of a *CACNA1C* variant and symptoms overlapping the original features of TS. In most individuals, a variant was identified using a cardiac gene panel (typically 40–50 genes); contributions of potential variants in other genes cannot be assessed. Whole exome analysis was performed in a few cases.

Case counts and relative proportions of each variant were obtained from published (*n* = 68) and unpublished individuals (Fig. 1). To avoid duplication of case counts, survey respondents were asked to report whether their case was published and to include the citation. If it was unclear whether a case was a duplicate, direct contact was made with contributing authors. Symptom occurrence reported in the tables herein included only data obtained from survey responses because of the relative incompleteness of information provided in case reports, as these publications focus mostly on cardiac presentations of neonates and generally do not include any information on extra-cardiac symptoms or the constellation of symptoms that may present later in childhood.

In order to facilitate comparison across reportedly different phenotypes, the study participants were organized into 5 separate categories (Fig. 1). *A*: 30 survey participants reported the Gly406Arg variant in exon 8A, comprising the TS1 group. *B*: 11 survey participants reported the Gly406Arg variant in exon 8. This group is identified as TS2 Gly406Arg. *C*: 6 survey participants harbored the Gly402Ser variant in exon 8. While this group was historically included within TS2, we choose to analyze these patients separately to compare and contrast the effects of distinct amino acid changes vs. exon locus. *D*:12 survey respondents reported a diagnosis of LQT8 only, with a variety of distinct *CACNA1C* variants across this group. In order to evaluate whether these patients truly represent a cardiac-selective phenotype, we group these participants as diagnosed nsLQT8. *E*: The final category represents all participants who reported a *CACNA1C* variant but do not fall within one of the other four categories (28 survey respondents). Among these, no more than two participants reported the same amino acid change.

### Survey

To characterize the natural history of TS, a 150-question survey was administered via Google Forms to collect information on symptoms associated with organ systems known to be affected, treatments, and outcome. For reference, the questionnaire is provided as Additional file 1. The survey also included questions about pregnancy and prenatal concerns in the mothers. The majority of respondents were the parents/guardians who completed the survey on behalf of their TS children; several adults (largely individuals diagnosed with nsLQT8) answered for themselves. No medical records were requested. Responses were collected after informed consent and the study was approved by the Institutional Review Board of Genetic Alliance (Washington, DC, USA).

Not all questions were answered by each participant. We did not assume that a non-answer was the equivalent of “No,” and thus the sample size for each question reflects the total number of respondents for that question.

Statistical analysis evaluating whether the results across all categories were associated were done using the Fisher Exact test and applied to major phenotype groups with greater numbers to improve the power of the statistical results.

## Results

### Study population

#### Overall survey yield

Of the 105 individuals invited to participate in the survey, 87 submitted a consent form and completed the survey. Of those 87, 28 had also been published previously as case reports that were focused primarily on cardiac presentations (Fig. 1). These individuals were able to provide additional information on extra-cardiac phenotypes via the survey. Affected individuals represented in the survey data range from 1 to 71 years of age, with an average age of 13.8 among 71 living individuals. Many of the surveyed individuals carried *CACNA1C* gene variants different from the canonical TS1 and TS2 variants, but nonetheless shared some TS phenotypes. Several patients also contain a frameshift or deletion in *CACNA1C* rather than a missense variant (Table 1: Genotypes of study population).

**Table 1:** Genotypes of study population

TS1 Gly406Arg		TS2 Gly406Arg		Gly402Ser		Diagnosed nsLQT8		Other Missense Variants		Other Frameshifts/Deletions	
Variant	n	Variant	n	Variant	n	Variant	n	Variant	n	Variant	n
Gly406Arg Exon 8A	30	Gly406Arg Exon 8	11	Gly402Ser Exon 8	6	Ala28Thr	1	Val403Met	1	Glu2062Term	1
						Leu207Arg	1	Ser405Arg	2	c.2089 + 1G > T	1
						Ile304Thr	2	Glu407Gly	1	Gly1173_Gln1175del	1
						Arg518Cys/ His	3	Gly419Arg	2	12p13.33p13.32(191,619-4,650,065)x1	1
						Arg858His	3	Leu658Pro	1	12p13.33(2,083,121-2,673,575)x3	1
						Glu771Lys	2	Val618Gly	1		
								Gly705Glu	1		
								Arg858His	1		
								Ala1012Val	1		
								Cys1021Arg	2		
								Gly1047Arg	1		
								Glu1115Lys	1		
								Ile1166Thr	2		
								Val1167Ala	1		
								Val1363Leu/Met	2		
								Val1411Leu	1		
								Ala1473Gly	1		
								Ala1521Pro	1		
**Total**:	**30**	**Total**:	**11**	**Total**:	**6**	**Total**:	**12**	**Total**:	**23**	**Total**:	**5**

#### Participants with TS1

Survey responses were obtained for 30 individuals with TS1 (14 female and 16 male), 16 of whom had been identified previously in case reports and 14 who were not previously reported (Fig. 1). We also identified 18 case reports of genetically-confirmed individuals, and 4 non-confirmed cases included in the Splawski et al., 2004 publication [10], for which no parental surveys were acquired. Hence, 48 genetically-confirmed Gly406Arg (exon 8A) individuals were identified, 34 of whom included in this study were previously reported (some individuals were included in more than one published report) [10, 18–32].

#### Participants with TS2

Survey responses were obtained from 11 individuals with TS2 who harbor the Gly406Arg variant within exon 8 (5 female and 6 male), including 3 individuals previously identified in case reports [13, 28, 33, 34]. Two additional individuals were reported solely in the literature but did not complete a survey [13, 34], resulting in 13 individuals known to have TS2 (Gly406Arg, Exon 8) (Fig. 1).

Another variant, Gly402Ser (exon 8), was originally classified as TS2 in a single child [13]. We collected 6 surveys from individuals with this variant. All 6 cases in our cohort are living. However, as more individuals have been identified with this specific variant, it has become clear that this variant produces a distinct phenotype from TS2 Gly406Arg. We therefore describe this variant separately.

#### Participants with additional variants in CACNA1C

**Fig. 2:**
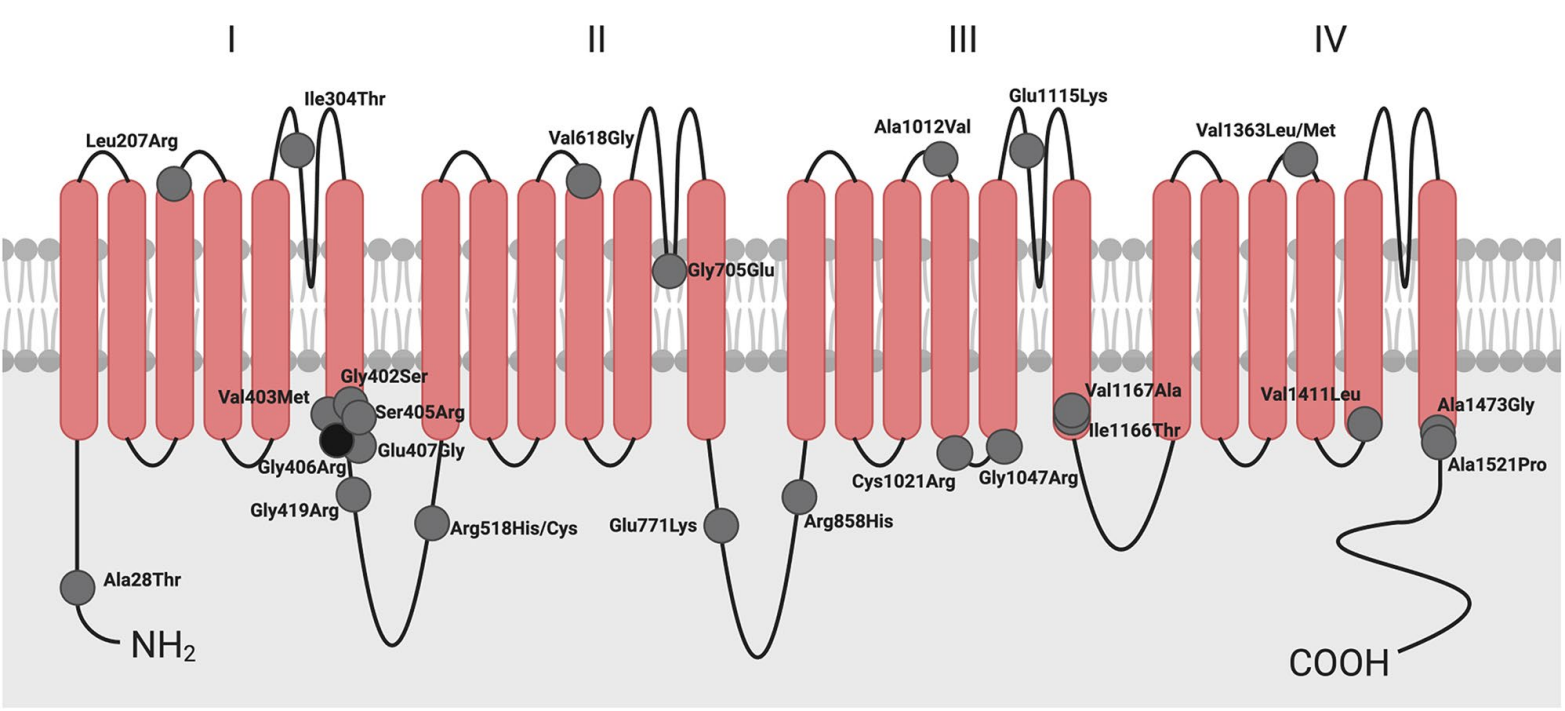
Schematic of the Ca_V_1.2 pore forming subunit showing the locus of mutations described within this study. Image created with BioRender.com

We collected surveys for 40 individuals with a range of molecular changes in *CACNA1C* (Fig. 2). One group was a cohort of individuals diagnosed with only LQT8. There is extensive literature on *CACNA1C* variants that selectively cause LQT8 [for review, see [35] that suggests individuals with these variants may not have extra-cardiac phenotypes common to individuals diagnosed with TS. In our survey, nine nsLQT8 families encompassing 12 individuals responded. We analyzed these individuals as a separate group, designated ‘diagnosed nsLQT8’, in order to evaluate the hypothesis that these patients do not harbor any extra-cardiac symptoms. Our analysis includes only variants noted in survey responses, although additional variants have been reported in the literature (Table 1: Genotypes of study population).

The remaining 28 respondents with additional variants in *CACNA1C* carry a variety of diagnoses, such as atypical TS or *CACNA1C*-related disorder. In the present study, we analyze these respondents as a single combined group.

#### Average cohort ages & average ages of deaths

The original 2004 TS phenotypic characterization reported the known average age of death of 17 TS1 children at 2.5 years [10]. Survival ages were not reported in that study because of the small number and young ages of the children. Of the 18 literature-reported TS1 children, nine of 18 (50%) died by the time of publication of their respective reports. The calculated average age of these combined literature-reported deaths was 1.13 years (range of death 1–31 months), lower than the original average age of death, possibly due to earlier diagnosis. For the other nine literature-reported individuals, survival status was not reported.

Of the thirty TS1 children in our cohort, 8/30 (27%) have died, with an average age at death of 3.2 years (age range of death 3 months-6 years). Seven of these eight were male children. The average age of living participants is 11.54 years (age range 1–30 years). The average age of living males is 14 years (range 5–22 years) and of females, 13 years (range 1–30 years) (Table 2: Average age of death and surviving cohort).

The original description of TS2 reported on one TS2 Gly406Arg child who died after publication at 6 years of age [13]. A second report described a TS2 Gly406Arg child who died at 52 days [34]. Of the 11 TS2 Gly406Arg individuals for whom surveys were obtained, three have died at 3 months, 5 months, and 10 years of age. The average age of the surviving 8 TS2 Gly406Arg children in our cohort is 7.5 years (age range 2–16 years) (Table 2: Average age of death and surviving cohort).

Individuals with the Gly402Ser variants in our study averaged 12.17 years of age, ranging from 10 to 15 years and did not include any individuals who were deceased. In all cases for which exon locus was reported, the variant occurred within exon 8, corresponding to the original description of TS2. All individuals with a nsLQT8 diagnosis were living, averaging 28 years of age (range 6–71 years). Finally, for other *CACNA1C* variants, the 23 living individuals averaged 11.2 years of age (range 1–42 years). 5 individuals died at ages averaging 6.08 years at death (range 1.67-13 years).

**Table 2:** Average age of death and surviving cohort

	TS1 Gly406Arg (*n* = 30)	TS2 Gly402Arg (*n* = 11)	Gly402Ser (*n* = 6)	Diagnosed nsLQT8 (*n* = 12)	Other (*n* = 28)
Total Living	22	8	6	12	23
Average Age, Living (years)	11.54	7.5	12.17	28	11.2
Range, Living (years)	1–30	2–16	10–15	6–71	1–42
Total Deceased	8	3	0	0	5
Average Age, Death (years)	3.18	3.57	---	---	6.08
Range, Death (years)	0.25-6	0.25-10	---	---	1.67-13

### Cardiac presentations from gestation through early childhood

#### Prenatal presentations

In our TS1 cohort, 13/30 (43%) fetuses had cardiac presentations *in utero*, all presenting with fetal bradycardia. In 8 of these patients (8/13, 62%), the bradycardia was identified as secondary to 2:1 atrioventricular block (AVB). The earliest recognition of these arrhythmias was at 19 weeks gestation (Table 3: Early development – cardiac concerns).

Caesarean births were frequent among pregnancies with TS1 fetuses, occurring in 18/30 (60%), with a range of fetal gestational age at birth of 28–41 weeks. Of these caesarean births, half were delivered pre-term (before gestation week 37) (9/18, 50%). Three additional pre-term TS1 births occurred via vaginal delivery.

Electrocardiographic abnormalities were present at birth in 18/30 (60%) of TS1 newborns (13 LQT, 15 bradycardia, and 6 with 2:1 AVB). Arrhythmias were detected later in infancy or childhood in additional children; 9 had arrhythmias during surgery, and 2 (18%) suffered unprotected cardiac arrest at age 3 and 6 years of age, prompting their diagnosis (Table 3: Early development – cardiac concerns).

Among the 18 TS1 literature-only cases, prenatal cardiac concerns were discussed in 6 cases [10, 18–32]. Pre-term Caesarean births were performed due to bradycardia (5/6, 83%) or 2:1 AVB (3/6. 50%).

TS2 Gly406Arg surveys indicate 7/11 cases (64%) had cardiac presentations *in utero* (5 bradycardia and 4 2:1 AVB). Caesarean births were also common among pregnancies with TS2 Gly406Arg individuals: 9/11 (82%) of individuals were delivered by Caesarean section, and 4/9 of these caesarean births were pre-term (44%). One additional individual pre-term birth occurred via vaginal delivery. At birth, 9/11 (82%) TS2 infants were noted to have cardiac concerns (4 LQT, 8 bradycardia, and 7 with 2:1 AVB) (Table 3: Early development – cardiac concerns).

In contrast, none of the Gly402Ser children presented *in utero* or at birth with any cardiac manifestations, nor were any delivered pre-term or by Cesarean section. None of the individuals with a diagnosis of nsLQT8 had cardiac concerns *in utero* or were born pre-term, but 3/12 were delivered by Caesarean section. For those with other variants in *CACNA1C*, 5/28 (18%) were identified to have cardiac concerns *in utero*. Five of 27 (19%) were born pre-term, and several were delivered by Caesarean section. (9/27, 33%) (Table 3: Early development – cardiac concerns).

**Table 3:** Early development – cardiac concerns

	TS1 Gly406Arg yes/total (%) *n* = 30	TS2 Gly406Arg yes/total (%) *n* = 11	Gly402Ser yes/total (%) *n* = 6	Diagnosed nsLQT8 yes/total (%) *n* = 12	Other yes/total (%) *n* = 28	Correlation *p*-value
Cardiac Problems Before Birth	13/30 (43)	7/11 (64)	0/6 (0)	0/10 (0)	5/28 (18)	0.0012
Bradycardia	13/13 (100)	5/7 (71)	---	---	2/28 (7)	
2:1 AVB	8/13 (62)	4/7 (57)	---	---	1/28 (4)	
Pre-term birth (< 37 weeks)	12/29 (41)	5/11 (45)	0/6 (0)	0/10 (0)	5/27 (19)	
Caesarean Birth	18/30 (60)	9/11 (82)	0/6 (0)	3/12 (25)	9/27 (33)	
Pre-term (< 37 weeks)	9/18 (50)	4/9 (44)	---	0/3 (0)	3/9 (33)	
Gestational age range (weeks)	28–41	32–40	39–42	39–42	34–41	
Cardiac problems at birth	18/30 (60)	9/11 (82)	0/6	1/6 (17)	13/23 (57)	0.253
LQT	13/29 (45)	4/6 (67)	---	0/6 (0)	8/23 (61)	0.0290
Bradycardia	15/29 (52)	8/10 (80)	---	1/6 (17)	5/23 (22)	
2:1 AVB	6/27 (22)	7/9 (78)	---	0/6 (0)	2/23 (9)	
Diagnosed LQT	26/26 (100)	8/8 (100)	4/5 (80)	6/6 (100)	19/21 (90)	0.1925
Congenital heart defects	11/30 (37)	2/11 (18)	0/6	2/8 (25)	11/24 (46)	0.1986
ToF	2/30 (7)	0/11 (0)	---	0/2 (0)	1/24 (4)	
PDA	7/30 (23)	2/11 (18)	---	0/2 (0)	5/24 (21)	
PFO	3/30 (10)	0/11 (0)	---	0/2 (0)	1/24 (4)	
ASD	0/30 (0)	0/11 (0)	---	0/2 (0)	2/24 (8)	
VSD	2/30 (6)	1/11 (9)	---	0/2 (0)	2/24 (8)	
HCM	2/30 (6)	1/11 (9)	---	0/2 (0)	1/24 (4)	
Other	8/30 (27)	1/11 (9)	---	0/2 (0)	5/24 (21)	
Arrythmias	30/30 (100)	9/11 (82)	4/6 (67)	10/11 (91)	13/16 (81)	0.168
Triggers:						
At rest	0/28 (0)	0/9 (0)	0/6 (0)	0/10 (0)	0/13 (0)	
Hypoglycemia	6/28 (21)	2/9 (22)	0/6 (0)	1/10 (10)	2/13 (15)	
Infections	4/28 (14)	2/9 (22)	1/4 (25)	3/10 (30)	10/13 (77)	
Loud noise	4/28 (14)	0/9 (0)	0/4 (0)	2/10 (20)	4/13 (30)	
Dehydration	4/28 (14)	0/9 (0)	0/4 (0)	2/10 (20)	6/13 (46)	
Activity/excitement	6/28 (21)	1/9 (11)	1/4 (25)	2/10 (20)	5/13 (38)	
Sleep	2/28 (7)	2/9 (22)	3/4 (75)	1/10 (10)	6/13 (46)	
Abnormal anesthesia response	19/28 (68)	7/9 (78)	3/6 (50)	5/10 (50)	4/13 (31)	0.1402
Sudden death	8/30 (27)	3/11 (27)	0/6 (0)	0/12 (0)	3/5 (60)	0.0333
Associated factors:						
Anesthesia	1/8 (13)	0/3 (0)	---	---	0/3 (0)	
Hypoglycemia	2/8 (25)	0/3 (0)	---	---	0/3 (0)	
Respiratory arrest	1/8 (13)	1/3 (33)	---	---	2/3 (67)	
Infection	2/8 (25)	0/3 (0)	---	---	0/3 (0)	
Unknown	3/8 (38)	1/3 (33)	---	---	0/3 (0)	
Small veins	9/14 (64)	4/5 (80)	1/5 (80)	---	8/19 (42)	

#### Infancy to early childhood

Of the TS1 infants, when cardiac electrical concerns were noted, echocardiograms (echo) were performed near birth and often revealed congenital heart defects (CHDs) (11/30 cases, 37%), including patent ductus arteriosus (PDA), patent foramen ovale (PFO), ventricular septal defect (VSD), hypertrophic cardiomyopathy (HCM), and Tetralogy of Fallot (ToF). Eight respondents noted CHDs but were not specific in naming the defect. Thirteen of 29 survey respondents (45%) indicated that LQT was diagnosed at or near birth (Table 3: Early development – cardiac concerns).

Of the TS2 Gly406Arg children, 10/11 also had neonatal echo for detection of CHDs, and 2/10 (20%) children were identified as having one or more cardiac defects, such as PDA, VSD, and left ventricular hypertrophy (LVH) (Table 3: Early development – cardiac concerns).

All Gly402Ser respondents appeared normal as infants with no apparent health concerns including no reported CHDs. The first cardiac presentation for all these children was sudden cardiac arrest (SCA) (average age 3.7 years, range 3–5 years) which they survived with varying degrees of sequelae. Activities precipitating the Gly402Ser SCA events varied, ranging from physical activity, excitement, sitting quietly, or even sleeping. Upon cardiac evaluations obtained post SCA, all the children were noted to have a prolonged QT interval (average = 511.5ms, range 460-650ms). Of the six children whose families completed a survey, none reported apparent or recognizable signs of risk in the immediate family or extended family pedigree indicative of SUD, syncope, or cardiac concerns. Subsequent genetic testing suggested that 83% (5/6) of individuals acquired their variant *de novo* (Table 3: Early development – cardiac concerns).

In those diagnosed with nsLQT8, only 1/6 (17%) were identified to have an arrhythmia at birth, which was bradycardia. Two of 8 respondents (25%) diagnosed with nsLQT8 noted having a CHD, but were not specific in naming the defect. In the individuals with other *CACNA1C* variants, 13/23 (57%) were diagnosed with cardiac electrical concerns at birth. 11/24 were identified as having CHDs, including ToF, PDA, PFO, atrial septal defect, VSD, and HCM (Table 3: Early development – cardiac concerns).

### Systematic evaluation of symptoms in TS

#### Sensitivity to anesthesia

Sensitivity to anesthesia is a serious threat to all TS individuals. Many children have experienced cardiac arrythmias upon administration of anesthesia (19/28 TS1 and 7/9 TS2 Gly406Arg) for correction of syndactyly, hip dysplasia, or dental procedures to remove diseased teeth [31]. In at least 12 TS1 cases (12/30; 40%), anesthesia was associated with the induction of ventricular arrhythmias during surgery. One TS1 child died after anesthesia administration. It is unclear whether hypoglycemia plays a role in this sensitivity, given that fasting is required prior to most surgical procedures. Half of Gly402Ser and diagnosed nsLQT8 individuals reported abnormal responses to anesthesia (3/6 and 5/10 respectively). Anesthesia is a risk for individuals with other variants as well, as 4/13 (31%) of these respondents reported an abnormal response to anesthesia (Table 3: Early development – cardiac concerns).

#### Hypoglycemia

While episodes of severe hypoglycemia were noted in the original description of TS [10]; their potential to cause serious adverse outcomes became apparent after the death of a 5-year-old hypoglycemic child who did not have an ICD-documented arrhythmic event. Survey results indicated that hypoglycemia is a common concern for TS1 children. Fourteen of 26 (54%) TS1 individuals were reported as having had at least one known episode of hypoglycemia at some time in life. Six of the 14 reported that their child was hypoglycemic at birth (46%); the other eight parents reported at least one hypoglycemic event during childhood. Five of these eight children had two or more hypoglycemic episodes. Hypoglycemia also affects TS2 Gly406Arg individuals. For these children, 3/11 (27%) experienced an episode of hypoglycemia at birth. One of these three had at least two additional hypoglycemic episodes during childhood (Table 4: Hypoglycemia and endocrine dysfunction).

Hypoglycemia was noted in two Gly402Ser individuals (2/4; 50%), and 4 of 11 (36%) survey respondents with diagnosed nsLQT8 reported at least one hypoglycemia episode, highlighting the potential for non-cardiac symptoms to arise in this category of patients. Many respondents in the other variant class (10/26; 38%) were affected, with 3 of these individuals reporting hypoglycemia at birth. Overall, hypoglycemia should be considered a serious life-threatening condition for all individuals with variants in *CACNA1C*.

**Table 4:** Hypoglycemia and endocrine dysfunction

	TS1 Gly406Arg yes/total (%) *n* = 30	TS2 Gly406Arg yes/total (%) *n* = 11	Gly402Ser yes/total (%) *n* = 6	Diagnosed nsLQT8 yes/total (%) *n* = 12	Other yes/total (%) *n* = 28	Correlation *p*-value
Hypoglycemia	14/26 (54)	3/11 (27)	2/4 (50)	4/11 (36)	10/26 (38)	0.6123
Hypoglycemic at birth	6/14 (43)	3/11 (27)	0/6 (0)	0/6 (0)	3/3 (100)	
Hypoglycemic death	2/7 (29)	0/1 (0)	0/6 (0)	0/11 (0)	---	
Pituitary Gland						
Small birth size	12/30 (40)	4/10 (40)	0/6 (0)	1/11 (9)	4/5 (80)	
Growth hormone replacement	2/29 (7)	1/10 (10)	0/6 (0)	---	0/6 (0)	
Early puberty	0/6 (0)	2/5 (40)	---	0/11 (0)	0/10 (0)	
Thyroid						
Overweight	3/30 (10)	1/9 (11)	1/6 (17)	1/11 (9)	4/26 (15)	
Underweight	5/30 (17)	6/9 (67)	0/6 (0)	2/11 (18)	7/26 (27)	
Adrenal/pineal glands						
Problems sleeping	12/29 (41)	6/10 (60)	2/6 (33)	4/11 (36)	13/23 (57)	
Nightmares	6/20 (30)	0/5 (0)	1/6 (17)	2/7 (29)	5/18 (28)	

#### Endocrine concerns

Forty percent (12/30) of TS1 newborns were considered small for gestational age (SGA). 31% (9/29) of TS1 children remained underweight into childhood, and two required growth hormone therapy early in childhood (Table 4: Hypoglycemia and endocrine dysfunction). 40% (4/10) of TS2 Gly406Arg infants were considered SGA with 50% (5/10) remaining underweight into childhood; one required growth hormone therapy.

Although most TS children were too young to determine if precocious puberty was of concern, 6/6 TS1 individuals have reached puberty within the average range (8–14 years of age), while two female TS2 Gly406Arg 7-year-olds are being evaluated for precocious puberty. This was not a problem reported by any respondents diagnosed with nsLQT8 (0/11) or other respondents (0/10). Sleep disturbances were noted in 12/29 (41%) TS1, 6/10 (60%) TS2 Gly406Arg, and 2/6 (33%) Gly402Ser children. Problems sleeping were shared by those diagnosed with nsLQT8 and the other variant respondents, affecting 4/11 (36%) and 13/23 (57%) respectively (Table 4: Hypoglycemia and endocrine dysfunction).

#### Pulmonary/respiratory concerns

Pulmonary dysfunction has been reported in TS children [10]. Survey data revealed pulmonary complications in TS1 and TS2 Gly406Arg infants at birth and during infancy, with 10/24 (42%) of TS1 infants experiencing pulmonary problems, some associated with PDA. One TS2 Gly406Arg infant without PDA had primary pulmonary hypertension with right-to- left atrial level shunting and died at 3 months of age due to hypoxic arrest (despite pulmonary vasodilator medications) without documentation of arrhythmia. Pulmonary presentations persisted throughout childhood in TS1 with 10/21 (48%) respondents reporting frequent pneumonias. Interestingly, 8/10 TS1 cases who reported frequent pneumonias were male. Other pulmonary concerns in the TS1 children included asthma and the need for nebulizer treatments for colds and infections. Three of 6 (50%) TS2 Gly406Arg children also suffered frequent pneumonias. However, respiratory concerns were not commonly reported in Gly402Ser individuals (Table 5: Respiratory and immune dysfunction).

Three out of 4 (75%) of those diagnosed with nsLQT8 reported respiratory concerns in early childhood. Survey respondents with other variants noted respiratory concerns in early childhood (8/10, 80%), most commonly frequent pneumonia (6/10, 60%). Additionally, two individuals died from respiratory arrest: one from respiratory syncytial virus requiring O_2_ at time of death and one from respiratory failure. An additional individual had a chest infection at death requiring an x-ray and suffered cardiac arrest during the x-ray. This suggests that respiratory concerns and infection are common and potentially life-threatening among individuals harboring a *CACNA1C* variant.

**Table 5:** Respiratory and immune dysfunction

	TS1 Gly406Arg yes/total (%) *n* = 30	TS2 Gly406Arg yes/total (%) *n* = 11	Gly402Ser yes/total (%) *n* = 6	Diagnosed nsLQT8 yes/total (%) *n* = 12	Other yes/total (%) *n* = 28	Correlation *p*-value
Respiratory concerns	13/29 (45)	3/10 (30)	1/6 (17)	4/6 (67)	9/20 (45)	0.4695
Early childhood respiratory concerns:	10/24 (42)	2/10 (20)	0/6 (0)	3/4 (75)	8/10 (80)	
Aspiration	2/29 (7)	1/10 (10)	1/6 (17)	0/4 (0)	4/10 (40)	
Frequent pneumonia	10/21 (48)	3/6 (50)	1/6 (17)	0/4 (0)	6/10 (60)	
Asthma	7/23 (30)	1/9 (11)	0/6 (0)	3/4 (75)	1/10 (10)	
Nebulizer treatments	5/20 (25)	1/9 (11)	0/6 (0)	3/4 (75)	0/10 (0)	
CRAP use	1/6 (17)	---	---	---	---	
Respiratory deaths	1/7 (14)	1/2 (50)	---	---	2/3 (67)	
Frequent infections	9/25 (36)	5/11 (45)	0/6 (0)	9/11 (82)	11/21 (52)	0.0137
Delayed wound healing	2/30 (6)	1/9 (11)	0/6 (0)	2/9 (22)	4/19 (23)	0.0390

#### Immune system

Thirty-six percent (9/25) of TS1 respondents reported frequent, recurring infections. One TS1 individual was found to have IgG2 deficiency, presumed to be responsible for infectious complications of bronchopneumonia, bronchitis, obstructive lung disease and asthma. 45% (5/11) of TS2 Gly406Arg children reported having frequent infections, though the exact nature of these infections was unknown. While no Gly402Ser individuals had immunological concerns, those with other variants in *CACNA1C* reported frequent infections, including 9/11 (82%) respondents diagnosed with nsLQT8 and 11/21 (51%) of those within the other variant category. The extent of immune dysfunction in TS individuals is otherwise not well understood given that most have not undergone comprehensive testing for immune deficiencies, however it does appear to be a recurring feature across the patient population (Table 5: Respiratory and immune dysfunction).

#### Syndactyly

Syndactyly in combination with arrythmias are the most common characteristics of the TS1 infant [10] and are often recognized at birth. The syndactyly observed in TS1 newborns has been classified as simple (skin and soft tissue only) or complex (skin, soft tissue, and bone). 96% of TS1 cases (45/47, respondents and literature cases combined) had either hand and/or foot syndactyly, with at least 89% (42/47) of genotyped positive TS1 children having obvious syndactyly (one or both hands). Survey results of TS1 individuals indicated 26/30 (87%) had obvious hand syndactyly, while two additional children, upon closer examination, exhibited slight simple syndactyly, totaling 28/30 (93%) in our cohort (Table 6: Skeletal, facial, skin, and dental).

Syndactyly of 4th and 5th digit among TS1 children is an atypical presentation that differs from the typical fusion of the third and fourth finger. Our survey results identified that 87% (26/28) of TS1 children with obvious syndactyly presented with inclusion of the 4th and 5th digit, with the most frequent finger syndactyly including digits 3–5 (*n* = 15), 4–5 (*n* = 8) and 2–5 (*n* = 3). Of the 26 children with obvious syndactyly that included the 5th digit, 16/26 (62%) had bilateral involvement, with four (15%) having only left-hand 4–5 syndactyly and six (23%) having different finger involvement in each hand. The remaining 2 of 30 (7%) TS1 individuals have classic bilateral 3–4 syndactyly (Table 6: Skeletal, facial, skin, and dental).

Unlike TS1, TS2 has not been associated with syndactyly, allowing for phenotypic distinction between the two types of TS [10, 13]. Only one TS2 Gly406Arg child was noted as having syndactyly; however, this child has a secondary genetic change [4q31.3q32.1 (155,189,952 − 155,654,941)x1], which is sporadically associated with 5th digit abnormalities and webbing between fingers [36]. Likewise, no respondents with Gly402Ser or diagnosed with LQT8 reported any syndactyly of fingers or toes. Unfortunately, this lack of a recognizable physical anomaly may reduce identification of these infants at birth (Table 6: Skeletal, facial, skin, and dental).

Survey respondents in the other variant class fall in the middle, with 13/27 (48%) reporting finger syndactyly and 11/27 (41%) reporting toe syndactyly. Ten of the 13 with finger syndactyly have fusion of digits 4–5, matching the TS1 hallmark.

**Table 6:** Skeletal, facial, skin, and dental

	TS1 Gly406Arg yes/total (%) *n* = 30	TS2 Gly406Arg yes/total (%) *n* = 11	Gly402Ser yes/total (%) *n* = 6	Diagnosed nsLQT8 yes/total (%) *n* = 12	Other yes/total (%) *n* = 28	Correlation *p*-value
Bone development abnormalities	28/30 (93)	6/11 (55)	0/6 (0)	0/11 (0)	16/27 (59)	0.0054
Finger syndactyly	28/30 (93)	1/11 (9)	0/6 (0)	0/11 (0)	13/27 (48)	< 0.0001
Includes 4–5	26/28 (93)	1/11 (9)	---	---	10/27 (37)	
Bilateral	18/28 (64)	1/11 (9)	---	---	7/27 (26)	
Toe syndactyly	24/30 (80)	1/11 (9)	0/6 (0)	0/10 (0)	11/27 (41)	
2–3 toe	23/24 (96)	1/11 (9)	---	---	10/27 (37)	
Bilateral	23/24 (96)	1/11 (9)	---	---	10/27 (27)	
Hip dysplasia	1/30 (3)	3/9 (33)	0/6 (0)	0/3 (0)	2/17 (12)	
Joint contractors	0/30 (0)	6/8 (75)	0/6 (0)	0/3 (0)	0/17 (0)	
Club foot	0/30 (0)	3/11 (27)	0/6 (0)	0/3 (0)	1/17 (6)	
Facial abnormalities	26/27 (96)	8/8 (100)	6/6 (100)	3/3 (100)	20/21 (95)	> 0.9999
Round face	23/25 (92)	7/9 (78)	3/6 (50)	2/3 (67)	12/21 (57)	
Broad forehead	23/23 (100)	7/9 (78)	4/6 (67)	---	5/21 (29)	
Thin upper lip	22/22 (100)	7/9 (78)	6/6 (100)	0/3 (0)	2/21 (13)	
Depressed maxillary	5/23 (22)	0/7 (0)	0/6 (0)	0/3 (0)	0/21 (0)	
Low set ears	11/22 (50)	7/9 (78)	0/6 (0)	1/3 (33)	8/21 (38)	
Flat bridge nose	6/15 (40)	2/4 (50)	1/6 (17)	0/3 (0)	7/21 (33)	
Hypertelorism	10/22 (45)	---	0/6 (0)	---	1/21 (5)	
Flat philtrum	6/21 (29)	1/9 (11)	0/6 (0)	0/3 (0)	0/21 (0)	
Bald at birth	26/27 (96)	2/11 (18)	1/6 (17)	1/3 (33)	11/21 (52)	
Skin abnormalities	26/27 (96)	6/11 (55)	3/6 (50)	7/7 (100)	9/12 (75)	0.0025
Skin sloughing	7/20 (35)	2/2 (100)	0/6 (0)	1/7 (14)	5/12 (42)	
Odd color	9/20 (45)	1/5 (20)	1/6 (17)	2/7 (29)	1/12 (8)	
Hot/cold sensitivity	26/27 (96)	2/11 (18)	3/6 (50)	5/7 (71)	9/12 (75)	
Dental abnormalities	16/22 (73)	7/7 (100)	2/6 (33)	9/9 (100)	20/21 (95)	0.0029
Frequent cavities	9/22 (41)	3/5 (60)	0/6 (0)	3/9 (33)	7/21 (33)	
Small teeth	11/18 (61)	2/5 (40)	0/6 (0)	2/9 (22)	3/21 (14)	
Misplaced teeth	9/11 (82)	3/3 (100)	1/6 (17)	3/9 (33)	6/21 (29)	
Tooth extraction surgery	7/16 (44)	4/7 (57)	1/6 (17)	4/9 (44)	6/21 (29)	
Sedation concerns	4/9 (44)	2/2 (100)	---	0/9 (0)	1/21 (5)	

#### Bones and joints

TS2 Gly406Arg individuals are distinguished from TS1 by the presence of skeletal development abnormalities, namely hip dysplasia, which we identified in 3/9 (33%) TS2 Gly406Arg respondents. TS2 Gly406Arg children are also identified as having joint contractures of the hands and feet (6/8; 75%) and/or a club foot (3/11; 27%). Radial head dysplasia was noted in a pair of TS2 Gly406Arg twins and protrusion of the distal end of the sternum was noted in two children. Six TS2 Gly406Arg respondents also noted joint hypermobility in an open-ended request for additional concerns. These abnormalities were generally absent in TS1 individuals. Twenty-nine of 30 (97%) TS1 children lacked any bone abnormalities noted in TS2 Gly406Arg children (other than syndactyly), however, one TS1 child was noted to have hip dysplasia. No bone and joint concerns were reported by Gly402Ser or diagnosed nsLQT8 individuals. Bone and joint concerns were not common in patients harboring other variants, with just two individuals reporting hip dysplasia (2/17; 12%) and one with club foot (1/17; 5%) (Table 6: Skeletal, facial, skin, and dental).

#### Hair growth, macular-facial development

A round face is a common feature of TS1 children (92%, 23/25) and this facial shape generally remains into adult life (5 of 6 TS1 surviving adults have retained the appearance of a round, infantile face). Round faces are also common in TS2 children (78%, 7/9). A broad forehead or a receding hairline is noted in all TS1 children and in 78% (7/9) of TS2 Gly406Arg children. Another hallmark facial feature noted in 22/22 (100%) TS1 children and 7/9 (78%) TS2 Gly406Arg children is a thin upper lip. In TS1, 6/21 (29%) have a flat philtrum, particularly recognized when smiling; this appears to be an infrequent characteristic in TS2 Gly406Arg individuals (1/9, 11%). A depressed maxilla or a hypertrophied mandible is noted in 5/23 (22%) TS1 children, but this facial feature was not recognized in any (0/7) of TS2 Gly406Arg children. Low set ears are noted in 11/22 (50%) TS1 and 7/9 (78%) TS2 Gly406Arg children. A flat nasal bridge was reported in 6 of 15 (40%) TS1 and 2 of 4 (50%) TS2 Gly406Arg children (Table 6: Skeletal, facial, skin, and dental).

Gly402Ser individuals also had several of these features: three had round faces (50%), four had broad foreheads (67%), all six had thin upper lips, one had low set ears (17%), but none had a flat philtrum or depressed maxillary. Two diagnosed nsLQT8 patients reported facial abnormalities, however only 3 of the 12 survey respondents answered this question, limiting conclusions. Among the other variant respondents, a round face and low set ears were most commonly reported (12/21; 57% and 8/21; 38%, respectively). Thus, facial abnormalities appear to be common among individuals with *CACNA1C* variants, with some differences in pattern across the different categories.

Ninety-six percent (26/27) of TS1 newborns were born bald or with sparse hair. Only 18% (2/11) of TS2 Gly406Arg newborns had little or no hair at birth. The Gly402Ser respondents were similar to the TS2 Gly406Arg group, with only 1 of 6 (17%) reporting little or no hair at birth. Other cases fell in the middle, with 33% (1/3) of those diagnosed with nsLQT8 and 52% (11/21) of other patients reporting baldness at birth.

####  Dermatologic abnormalities

Skin disorders were noted among individuals with *CACNA1C* variants across the different groups. No respondents reported having the autoimmune disease of psoriasis, however skin sloughing, unrelated to environmental or geographical living conditions, was reported by 7/20 (35%) TS1, 2/2 TS2 Gly406Arg, 1/7 (14%) diagnosed nsLQT8 and 5/12 (42%) other respondents. Odd, strange, or diagnostically undetermined skin color anomalies were reported by 9/20 (45%) TS1, 1/5 (20%) TS2 Gly406Arg, 1/6 (17%) Gly204Ser, 2/7 (29%) diagnosed nsLQT8, and 1/12 (8%) other respondents (Table 6: Skeletal, facial, skin, and dental).

#### Dental features

Forty-one percent (9/22) of TS1 children had frequent, large dental caries/decay, with 7 of 16 (44%) requiring surgical removal of multiple diseased teeth. Three of 5 TS2 Gly406Arg children also struggled with frequent cavities (60%). Small teeth were noted in 11 of 18 (61%) TS1 and 2 of 5 (40%) TS2 Gly406Arg children. Misplaced teeth were reported in 9/11 (82%) TS1 respondents and 3/3 TS2 Gly406Arg children. Individuals with Gly402Ser did not report frequent cavities or small teeth, but 1/6 (17%) reported misplaced teeth and subsequent corrective surgery. A few diagnosed nsLQT8 also reported dental concerns, including frequent cavities (3/9; 33%), misplaced teeth (3/9; 33%), and tooth extraction surgery (4/9; 44%). Other *CACNA1C* variant individuals reported frequent cavities (7/21; 33%), misplaced teeth (6/21; 29%), and tooth extraction surgery (6/21; 29%) (Table 6: Skeletal, facial, skin, and dental).

#### Skeletal muscle

Hypotonia is present in both TS1 (5/26, 19%) and TS2 Gly406Arg (5/7, 71%), but appears to be more frequent and severe in TS2 Gly406Arg, as evidenced by delay or impairment in walking. Only one Gly402Ser individual reported hypotonia (1/6; 17%). Although walking unassisted was delayed in some TS1 children, all children have learned to walk. Among survey respondents, all parents of TS2 Gly406Arg children considered their children to be lacking in physical coordination. Of note, one child requires mechanical assistance to walk, and two children are non-ambulatory at ages 6 and 10 years. As TS2 Gly406Arg children transition into older childhood, other muscular concerns become apparent. Many patients experience disproportionate upper versus lower body development (4/7; 57%) and body strength discrepancies between right and left side (3/5; 60%). In contrast, these characteristics were not reported by any TS1individuals. However, coordination issues were reported in TS1 (11/13; 61%), TS2 Gly406Arg (5/5, 100%), Gly402Ser children (2/6; 33%), diagnosed nsLQT8 (4/7; 57%), and other variant cases (11/19; 58%) (Table 7: Neuromuscular and sensory concerns).

**Table 7:** Neuromuscular and sensory concerns

	TS1 Gly406Arg yes/total (%) *n* = 30	TS2 Gly406Arg yes/total (%) *n* = 11	Gly402Ser yes/total (%) *n* = 6	Diagnosed nsLQT8 yes/total (%) *n* = 12	Other yes/total (%) *n* = 28	Correlation *p*-value
Neuro-muscular concerns	11/30 (37)	5/7 (71)	2/6 (33)	4/7 (57)	11/20 (55)	0.3977
Hypotonia	5/26 (19)	5/7 (71)	1/6 (17)	1/3 (33)	11/18 (61)	
Severe weakness	3/22 (14)	3/7 (43)	1/6 (17)	---	6/20 (30)	
Strength differences	0/28 (0)	3/5 (60)	0/6 (0)	---	---	
Uneven body growth	0/30 (0)	4/7 (57)	0/6 (0)	---	0/1 (0)	
Lack of coordination	11/13 (61)	5/5 (100)	2/6 (33)	4/7 (57)	11/19 (58)	
Delayed milestones	19/25 (76)	7/7 (100)	5/5 (100)	5/9 (56)	25/26 (96)	0.0213
Holding head (> 3mos)	11/18 (61)	3/3 (100)	1/3 (33)	5/9 (56)	11/20 (55)	
Sitting (> 6mos)	15/19 (79)	4/5 (80)	0/4 (0)	2/9 (22)	12/17 (71)	
Crawling (> 9mos)	14/18 (78)	4/4 (100)	0/3 (0)	1/9 (11)	12/14 (86)	
Standing (> 12mos)	15/20 (75)	7/7 (100)	1/5 (20)	1/9 (11)	9/15 (60)	
Walking (> 18mos)	10/20 (50)	5/6 (83)	5/5 (100)	1/9 (11)	8/17 (47)	
Running (> 24mos)	8/18 (44)	4/5 (80)	5/5 (100)	1/9 (11)	6/15 (40)	
Climbing (> 24mos)	8/16 (44)	5/6 (83)	5/5 (100)	2/8 (25)	8/14 (57)	
Jumping (> 30mos)	12/16 (67)	5/5 (100)	1/4 (25)	2/9 (22)	8/12 (67)	
Neurosensory concerns	20/30 (67)	8/11 (73)	4/6 (67)	11/11 (100)	23/27 (85)	0.0036
Diagnosed eye conditions	16/24 (67)	6/7 (86)	2/6 (33)	7/10 (70)	14/23 (61)	
Wears glasses	14/24 (58)	6/7 (86)	2/6 (33)	7/10 (70)	11/23 (48)	
Myopia	3/14 (21)	1/6 (17)	0/2 (0)	1/4 (25)	0/17 (0)	
Hyperopia	2/14 (14)	0/6 (0)	0/2 (0)	---	1/17 (6)	
Amblyopia	1/14 (7)	3/6 (50)	0/2 (0)	1/4 (25)	6/17 (35)	
Strabismus	3/14 (21)	2/6 (33)	1/2 (50)	0/4 (0)	2/17 (12)	
Astigmatism	3/14 (21)	0/6 (0)	0/2 (0)	4/4 (100)	6/17 (35)	
Retina Concern	2/14 (14)	0/6 (0)	0/2 (0)	0/4 (0)	0/17 (0)	
Cortical Blindness	0/30 (0)	1/7 (14)	0/6 (0)	0/4 (0)	1/17 (6)	
Diagnosed hearing concern	0/30 (0)	0/11 (0)	0/6 (0)	3/8 (38)	1/20 (5)	
Sounds irritate	11/25 (44)	5/10 (50)	2/6 (33)	7/11 (64)	13/21 (62)	
Taste concerns						
Food aversion	2/25 (8)	---	2/6 (33)	---	2/21 (10)	
Food textures	6/25 (19)	2/8 (25)	2/6 (33)	3/7 (43)	8/21 (38)	
Olfactory concern	0/30 (0)	0/9 (0)	1/6 (17)	---	0/1 (0)	
Tactile concern						
Clothing preferences	8/20 (40)	3/6 (50)	2/6 (83)	5/9 (56)	10/20 (50)	

#### Neurosensory

Many TS children have vision abnormalities requiring corrective lenses. 58% (14/24) of TS1 children wear corrective glasses for vision concerns including myopia (3/14), astigmatism (3/14), amblyopia (1/14), hyperopia (2/14), retina concerns (2/14), strabismus (3/14), and coloboma of the iris (1/14). Of 11 TS2 Gly406Arg respondents, 7 have had vision examinations. At the time of the survey, 6 required corrective lenses; 2 for strabismus, 3 for amblyopia, and 1 for myopia, while the remaining child was found to be cortically blind. Only 2/6 (33%) of Gly402S respondents had eye concerns, while glasses were commonly reported in LQT8 individuals (7/10; 70%). However, this higher percentage could reflect the older age of this cohort. 48% (11/23) of other *CACNA1C* variant individuals required glasses, with the most common visual concerns being amblyopia (6/17; 35%) and astigmatism (6/17; 35%) (Table 7: Neuromuscular and sensory concerns).

All TS1 and TS2 Gly406Arg and Gly402Ser children appeared to have normal or near normal hearing. The trend was similar in other variant individuals with only 1 individual reporting a diagnosed hearing concern (1/20; 5%). Hearing loss was most common in individuals diagnosed with nsLQT8 (3/8; 38%), though this could again reflect the older age of the cohort (Table 7: Neuromuscular and sensory concerns).

Sounds and particularly loud noises were reported as irritating and causing distress in 11 of 25 (44%) TS1 children, 5/10 (50%) TS2 children, and 2/6 (33%) Gly402S respondents. This was similar in the other variant group, with 62% (13/21) reporting irritation from loud noises (Table 7: Neuromuscular and sensory concerns).

#### Speech concerns

Sixty-nine percent (18/26) of TS1 parents surveyed considered their children to be speech delayed, and 6/9 (67%) TS2 Gly406Arg parents reported the same concern. Of the six TS2 Gly406Arg children considered delayed in speech, two are non-verbal (at ages 6 and 10), though one is adept at communicating with sign language and the written word on a laptop computer. Ten of 20 (50%) TS1 and 5/7 (71%) TS2 Gly406Arg children showed a delay in receptive speech understanding. Greater delays in expressive speech were reported for 13 of 20 (65%) TS1 and 5 of 7 (71%) TS2 Gly406Arg children. 20% (5/25) of TS1 children expressed themselves with odd sounds or had articulation disorders; 25% (2/8) of TS2 Gly406Arg children also exhibited similar articulation disorders. Speaking in full sentences was delayed in both TS1 and TS2 Gly406Arg individuals, as reported by parents. The average age at which TS1 children spoke in full sentences was 4.75 years (19/26, range 2.5-5 years). Only two TS2 children reported an age at which they spoke in full sentences, which averaged 4.75 years (range 2.5-7 years). Two out of 6 (33%) Gly402Ser respondents reported speech delay, which was similar for patients diagnosed with nsLQT8 (3/10; 30%). In contrast, speech delay was more common in other variant individuals (15/17; 88%) (Table 8: Neuropsychological characteristics and adult life skills).

**Table 8:** Neuropsychological characteristics and adult life skills

	TS1 Gly406Arg yes/total (%) *n* = 30	TS2 Gly406Arg yes/total (%) *n* = 11	Gly402Ser yes/total (%) *n* = 6	Diagnosed nsLQT8 yes/total (%) *n* = 12	Other yes/total (%) *n* = 28	Correlation *p*-value
Neurodevelopmental concerns Sspeech concerns	18/30 (60) 18/26 (69)	6/10 (60) 6/9 (67)	5/6 (83) 5/6 (83)	4/11 (36) 4/10 (40)	23/27 (85) 17/18 (94)	0.0314 0.0257
Speech delay	18/26 (69)	6/9 (67)	2/6 (33)	3/10 (30)	15/17 (88)	
Receptive delay	10/20 (50)	5/7 (71)	2/6 (33)	---	5/17 (29)	
Expressive delay	13/20 (65)	5/7 (71)	2/6 (33)	2/4 (50)	12/17 (71)	
Odd sounds	5/25 (20)	2/8 (25)	2/6 (33)	1/4 (25)	9/17 (53)	
Educational						
Global developmental delay	0/30 (0)	2/10 (20)	0/6 (0)	---	4/5 (80)	
Knows alphabet	13/17 (76)	1/4 (25)	6/6 (100)	12/12 (100)	9/20 (45)	
Age when spoke full sentences (yrs)	2.5–5	2.5–7	1–4	0–3.5	1.5–6	
At grade level Learning concerns:	5/17 (29) 11/16 (69)	0/5 (0) 4/4 (100)	0/1 (0) 4/6 (67)	9/11 (82) 4/9 (44)	3/15 (20) 12/15 (80)	
Problems reading	12/16 (75)	1/4 (25)	3/6 (50)	2/9 (22)	12/15 (80)	
Problems with numbers	10/15 (67)	4/4 (100)	3/6 (50)	4/9 (44)	10/15 (67)	
Accurate memory	8/18 (44)	1/4 (25)	1/4 (25)	2/9 (22)	2/15 (13)	
Delayed potty training (> 4yrs)	10/20 (50)	7/8 (86)	2/4 (50)	2/7 (29)	2/7 (29)	
Delayed night training (> 5yrs)	7/17 (41)	7/7 (100)	1/4 (25)	4/10 (40)	---	
Social ability						
Not tested	13/19 (68)	1/5 (20)	3/6 (22)	---	---	
Autism	6/12 (50)	4/9 (44)	1/3 (33)	2/8 (25)	8/14 (57)	0.6873
ADD/ADHD	5/11 (45)	5/9 (56)	3/5 (60)	1/8 (13)	4/14 (29)	0.2840
Shy/anxious	14/24 (58)	4/7 (57)	5/6 (83)	2/10 (2)	7/19 (37)	
Odd behaviors	13/23 (57)	4/5 (80)	1/4 (25)	2/10 (2)	6/19 (32)	
Plays with age group	12/23 (52)	2/4 (50)	1/4 (25)	6/10 (60)	8/19 (42)	
Prefers being alone	11/21 (52)	2/4 (50)	2/6 (33)	5/10 (50)	6/19 (32)	
Prefers quiet environment	14/21 (67)	3/5 (60)	2/6 (33)	3/10 (30)	7/19 (37)	
Prefers animals	8/19 (42)	1/5 (25)	0/6 (0)	3/10 (30)	4/19 (21)	
Other neural development	18/24 (75)	6/9 (67)	6/6 (100)	8/11 (73)	15/22 (68)	0.6462
Routine oriented	17/22 (77)	5/5 (100)	4/6 (67)	5/10 (50)	9/19 (47)	
Orders objects	9/23 (39)	1/3 (33)	1/4 (25)		6/19 (32)	
Seizure disorder	10/30 (33)	5/7 (71)	0/6 (0)	1/11 (9)	11/22 (50)	
Has phobias	9/24 (38)	0/4 (0)	0/6 (0)	2/8 (25)	1/14 (7)	
Anger/violence	11/23 (48)	2/5 (40)	2/6 (33)	1/8 (13)	4/14 (29)	
Depression	1/6 (17)	---	0/6 (0)	2/8 (25)	2/14 (14)	
OCD	4/19 (21)	---	1/6 (17)	2/8 (25)	3/14 (21)	
Schizophrenia	1/19 (5)	---	1/6 (17)	0/8 (0)	0/14 (0)	
Severe headaches	2/10 (11)	0/2 (0)	2/6 (33)	1/8 (13)	1/14 (7)	
Adult life skills						
Rides bus alone	2/6 (33)	---	---	6/6 (100)	---	
Has driver’s license	1/6 (17)	---	---	6/6 (100)	---	
Makes monetary change	1/6 (17)	---	---	6/6 (100)	---	

#### Neurodevelopment and educational/learning delays

The TS1 and TS2 Gly406Arg school age population is small, but most experience educational and learning delays. Thirteen of 17 (76%) of the TS1 children were able to learn the alphabet. Twelve of 16 (75%) TS1 and 1 of 4 (25%) TS2 Gly406Arg children have not attained age-appropriate reading level skills, and 10 of 15 (67%) TS1 and 4 of 4 TS2 Gly406Arg children lack numerical/computational skills. Three of 6 (50%) Gly402Ser children have problems with reading and math, although these delays are difficult to separate from potential effects of SCA events. Problems with reading and math skills were less prevalent in diagnosed nsLQT8 individuals (2/9; 22% and 4/9 22%, respectively), but common in individuals with other variants (12/15; 80% and 10/15; 67%, respectively). Delayed toilet training (> 4 years old) is noted in 10/20 (50%) of TS1 and 7/8 (86%) of TS2 Gly406Arg children. Nighttime training was delayed in TS1 (7/17, 41%) and TS2 Gly406Arg (7/7, 100%) children (Table 8: Neuropsychological characteristics and adult life skills).

Interestingly, 8/18 (44%) TS1 and 1/4 (25%) TS2 Gly406Arg children were described as having an “amazing and accurate memory” by parents. 6 of 12 (50%) TS1 survey respondents reported a diagnosis of autism, however this number is lower than earlier reports indicating as many as 80% of these patients suffer from Autism Spectrum Disorder [10]. Thus, it is possible that the relatively low number of respondents answering this question and the young age of many of the individuals contributed to the lower value. In addition, 5/11 (45%) TS1 respondents reported attention-deficit disorder (ADD) or attention-deficit/hyperactive disorder (ADHD). Of nine TS2 Gly406Arg respondents, all were diagnosed with either autism (4/9) or ADD/ADHD (5/9) (Table 8: Neuropsychological characteristics and adult life skills).

Seizure disorders were noted in 10/30 (33%) TS1 and 5/7 (71%) TS2 Gly406Arg individuals. Concerns of depression were seldom noted in TS1 (1/6, 17%), and were not reported in any TS2 Gly406Arg survey. 58% (14/24) of TS1 children were noted to be shy/anxious by their parents, as were 57% (4/7) of TS2 Gly406Arg children. 38% (9/24) of TS1 children were noted to have phobias, while this was not noted in TS2 Gly406Arg. Episodes of anger/violence were often noted in TS1 (11/23, 48%) and TS2 Gly406Arg (2/5, 40%) children. One TS1 individual has been diagnosed with schizophrenia (Table 8: Neuropsychological characteristics and adult life skills).

A few Gly402Ser children report psychosocial concerns, including 3/5 (60%) with diagnoses of ADD/ADHD and 1/3 (33%) diagnosed with autism. Each of these conditions was only noted in 1–2 individuals diagnosed with nsLQT8. Individuals with other variants reported autism and ADD/ADHD diagnoses (8/14; 57% and 4/14; 29%, respectively) and 50% (11/22) noted seizure disorders (Table 8: Neuropsychological characteristics and adult life skills).

#### Gastrointestinal and urinary systems

Gastrointestinal and urinary dysfunction have been noted as additional symptoms of TS as more individuals are living longer and are followed over time. Early in life, esophageal dysfunction manifested as dysphagia for 13/28 (46%) TS1 and 7/10 (70%) TS2 Gly406Arg children. Seven of 27 (26%) TS1 and 4/10 (40%) TS2 Gly406Arg respondents reported suffering from gastric reflux. Twelve of 29 (41%) TS1 and 6/10 (60%) TS2 Gly406Arg individuals reported severe gag reflex. Nasogastric tube use was reported for 9/25 (36%) TS1 and 5/10 (50%) TS2 Gly406Arg individuals. Frequent vomiting also occurred in 7/28 (25%) TS1 and 6/9 (67%) TS2 Gly406Arg individuals. Only 1 out of 6 (17%) Gly402Ser respondents noted any esophageal concerns, which were gastric reflux and frequent vomiting. Gastric reflux was common in diagnosed nsLQT8 and individuals with other variants, affecting 5/7 (71%) and 12/22 (55%) respectively (Table 9: Gastrointestinal and urinary dysfunction).

**Table 9:** Gastrointestinal and urinary dysfunction

	TS1 Gly406Arg yes/total (%) *n* = 30	TS2 Gly406Arg yes/total (%) *n* = 11	Gly402Ser yes/total (%) *n* = 6	Diagnosed nsLQT8 yes/total (%) *n* = 12	Other yes/total (%) *n* = 28	Correlation *p*-value
Esophageal concerns	19/30 (63)	8/10 (80)	3/6 (50)	7/7 (100)	21/22 (95)	0.0103
Difficulty swallowing	13/28 (46)	7/10 (70)	0/6 (0)	---	7/22 (32)	
Gastric reflux	7/27 (26)	4/10 (40)	1/6 (17)	5/7 (71)	12/22 (55)	
Frequent gagging	12/29 (41)	6/10 (60)	0/6 (0)	2/7 (29)	4/22 (18)	
Nasogastric tube	9/25 (36)	5/10 (50)	0/6 (0)	1/8 (13)	9/22 (41)	
Frequent vomiting	7/28 (25)	6/9 (67)	1/6 (17)	2/7 (29)	6/22 (27)	
Surgical procedures	2/30 (7)	---	---	---	---	
Stomach concerns	9/30 (30)	1/11 (9)	2/6 (33)	3/7 (43)	13/25 (52)	0.01274
Frequent stomach aches	9/27 (33)	1/3 (33)	2/4 (50)	3/7 (43)	12/25 (48)	
Omphalocele	0/30 (0)	1/11 (9)	0/6 (0)	---	0/5 (0)	
Ileal atresia/dilation	1/30 (3)	1/5 (20)	0/6 (0)	---	1/5 (20)	
Elimination concerns	12/28 (43)	6/10 (60)	5/6 (83)	7/7 (100)	22/25 (88)	0.0013
Constipation	11/19 (38)	6/10 (60)	5/6 (83)	7/7 (100)	21/25 (90)	
Diarrhea	2/28 (7)	2/10 (20)	0/4 (0)	4/7 (57)	4/25 (16)	
Urinary concerns	3/10 (30)	0/11 (0)	0/6 (0)	1/10 (10)	1/19 (5)	0.1372
Kidney abnormalities	3/10 (30)	0/11 (0)	0/6 (0)	1/10 (10)	1/19 (5)	
Body swelling	2/10 (20)	0/11 (0)	0/6 (0)	---	0/5 (0)	

Severe stomach aches were noted in 33% of TS1 (9/27) and TS2 Gly406Arg cases (1/3). Half of Gly402Ser respondents (2/4) and nearly half of those diagnosed with nsLQT8 (3/7; 43%) or other variants (12/25; 48%) also reported frequent severe stomach aches. Anatomic gastrointestinal defects that were reported included ileal atresia/dilation (1/ 30, 3% of TS1 children) and omphalocele with bowel malrotation (1/11 (9%) TS2 Gly406Arg children). These abnormalities required surgical repair.

Diarrhea has not been a major concern, affecting 2/28 children with TS1 (75) and 2/10 TS2 Gly406Arg children (20%). Constipation was more common across all groups, reported in 11 of 19 (38%) TS1 and 6 of 10 (60%) TS2 Gly406Arg children. Two male TS1 children reported receiving emergency room attention due to sustained penile pain and tumescence resulting from severe fecal blockage within the bowel. Penile pain is considered common in the pediatric population, resulting from several etiologies [37]. Severe constipation can exert external pressure on the bladder wall, causing referred pain to the genitalia. In the two male TS1 children who experienced this, emergency room assistance was required to remove the bowel blockage and relieve the pain. Constipation was common across TS groups, affecting 5/6 (83%) of Gly402Ser respondents, 7/7 (100%) diagnosed nsLQT8 and 21/25 (90%) other variant respondents.

As the TS1 population ages, there is growing evidence of kidney dysfunction (3/10; 30%). One 30-year-old TS1 woman was diagnosed with stage three kidney failure, ascribed to scarring associated with frequent urinary tract infections and severe constipation.

### Current therapies

#### Implantable cardiac defibrillators (ICDs)

Most of the surviving TS1 (24/30, 80%), TS2 Gly406Arg (9/11, 82%), Gly402Ser (6/6, 100%), and other *CACNA1C* variant (6/16, 38%) children reported having an ICD implanted early in life or soon after a cardiac event. Six TS1 individuals currently do not have ICDs due to their small size or parental decision; each family reported always having an AED with them. Currently two TS2 Gly406Arg children are managed with AEDs only. Of the 8/30 TS1 individuals who have died, 63% (5/8) were too young to consider ICD implantation. Although the other 3/8 had ICDs at the time of their deaths, ICD interrogation revealed no arrhythmias in two of the three. Of the three deaths among individuals with TS2 Gly406Arg (11 total), only one had an ICD at the time of death and interrogation revealed no precipitant arrhythmia. The causes of death of the other two children with TS2 Gly406Arg remain unknown. Two individuals with Gly402Ser have suffered multiple ICD shocks that have continued despite left cardiac stellate denervation (LCSD) surgery (see below) (Table 10: Cardiac medications and procedures).

**Table 10:** Cardiac medications and procedures

	TS1 Gly406Arg yes/total (%) *n* = 30	TS2 Gly406Arg yes/total (%) *n* = 11	Gly402Ser yes/total (%) *n* = 6	Diagnosed nsLQT8 yes/total (%) *n* = 12	Other yes/total (%) *n* = 28
Implanted Devices	22/30 (73)	9/11 (82)	6/6 (100)	0/12 (0)	7/26 (27)
Infant pacemaker	9/30 (30)	5/11 (45)	0/6 (0)	0/12 (0)	1/21 (10)
Infant ICD	7/30 (23)	1/11 (9)	0/6 (0)	0/12 (0)	0/26 (0)
Other ages ICD	17/30 (57)	8/8 (100)	6/6 (100)	---	6/16 (38)
Infant LCSD	3/30 (10)	1/11 (9)	0/6 (0)	---	1/5 (20)
Other ages LCSD	4/30 (13)	0/10 (0)	2/6 (33)	---	0/4 (0)
Beta-blockers	27/30 (93)	11/11 (100)	6/6 (100)	7/10 (70)	12/20 (60)
Propranolol	11/27 (41)	8/11 (72)	1/6 (17)	0/7 (0)	3/12 (25)
Inderal LA	1/27 (3)	0/11 (0)	0/6 (0)	1/7 (14)	0/12 (0)
Atenolol	4/27 (15)	0/11 (0)	0/6 (0)	0/7 (0)	1/12 (8)
Nadolol	5/27 (19)	3/11 (27)	4/6 (67)	6/7 (86)	6/12 (50)
Unknown	6/27 (22)	0/11 (0)	1/6 (17)	0/7 (0)	2/12 (17)
Additional medications	6/29 (21)	3/11 (27)	4/6 (67)	0/8 (0)	2/21 (10)
Mexiletine	5/29 (17)	3/11 (27)	4/6 (67)	0/8 (0)	2/21 (10)
Verapamil	1/29 (3)	0/11 (0)	0/6 (0)	0/8 (0)	1/15 (7)
Nifedipine	2/29 (7)	0/11 (0)	0/6 (0)	0/6 (0)	0/6 (0)

#### Beta-blockers

Twenty-seven of 30 TS1 individuals receive beta-blockers (BB) for management of Long QT syndrome (LQTS). In our survey cohort, the BBs prescribed include propranolol (11/27, 41%), atenolol (4/27, 15%), and nadolol (5/27, 19%). Of the three who did not take BBs, one child could not tolerate BB medications, a second child was prescribed verapamil, and a third was prescribed mexiletine only. Five of 19 (26%) TS1 individuals take BBs combined with mexiletine for arrhythmia control. One adult TS1 individual is on BBs and nifedipine. All 11 individuals in our TS2 Gly406Arg cohort take BBs. Most are prescribed propranolol (8/11, 72%), and the remaining children take nadolol (3/11, 27%). Three TS2 Gly406Arg children also take mexiletine in addition to a BB (3/11, 27%) (Table 10: Cardiac medications and procedures).

Children with the Gly402Ser variant all take BBs (6/6), most commonly nadolol (4/6, 67%), and one child takes propranolol (1/6, 17%). Four of 6 (67%) are also prescribed mexiletine. For diagnosed nsLQT8 patients, BBs are also common (7/10, 70%), with 6 of 7 (86%) taking nadolol and 1 individual prescribed Inderal LA. Individuals with other variants are commonly prescribed BBs (12/20, 60%), including propranolol (3/12, 25%), atenolol (1/12, 8%), and nadolol (6/12, 50%). Two of 21 patients also take mexiletine, and 1 is prescribed verapamil.

#### Left cardiac stellate denervation (LCSD)

The surgical procedure of left cardiac stellate denervation (LCSD) is sometimes undertaken to treat children with TS, despite high risk for arrhythmias under anesthesia. Typically, LCSD is performed when medication protocols are not tolerated or when uncontrolled adrenergically-driven arrhythmias are suspected. It is currently unclear if the TS or LQT8 arrhythmias are caused solely by adrenergic triggers since sleep and/or early morning arrhythmias are noted (Table 3: Early development – cardiac ﻿concerns). In our surveyed cohort, 7 of 30 (23%) TS1 children, 1 of 11 (9%) TS2 Gly406Arg children, and 2 of 6 (33%) Gly402Ser children have undergone LCSD. In TS1 children, 43% (3/7) underwent LCSD surgery in infancy (2–6 months), while 57% (4/7) received LCSD in childhood (17 months-8 years). The TS2 Gly406Arg child underwent LCSD at 2 months of age and the two Gly402Ser children underwent LCSD surgery in childhood (ages 9 and 10 years old). From the literature, we are aware of one additional TS1 [10] and TS2 Gly406Arg [13] child who underwent this procedure (Table 10: Cardiac medications and procedures).

The efficacy of LCSD in survival and control of arrhythmias, according to parental survey answers, indicates mixed results. Side effects of LCSD are generally minimal, though Horner’s syndrome is a common side effect [38]. Our survey assessed LCSD efficacy in arrhythmia prevention compared to pre-surgery. TS1, TS2 Gly406Arg and Gly402Ser children were all drug compliant before LCSD surgery. Survival rates with LCSD in TS1 is currently 57% (4/7), and 43% (3/7) have died since LCSD surgery. Three of seven (43%) TS1 children were prescribed maximum medication protocols but continued to have arrhythmia concerns; all three children have continued to have ICD shocks after LCSD surgery. The remaining 57% of TS1 individuals underwent LCSD surgery for added protection unrelated to ICD shocks, however 3 children died from non-arrhythmia events. One TS1 child did not tolerate beta-blocker medications and underwent LCSD for protected care but ICD shocks continued; subsequently, a right cardiac stellate denervation (RCSD) was also performed with no further ICD shocks to date. The one TS2 Gly406Arg child who received the surgery has died as a result of respiratory arrest without arrhythmia. The two diagnosed nsLQT8 Gly402Ser children are living, and have continued to endure ICD shocks but with reduced numbers as perceived by parents.

## Discussion

### Variable symptom presentation is universal among individuals with *CACNA1C* variants

A major theme emerging from the comparison of these different groups of patients is the multisystem nature of the disorder, even among patients previously diagnosed with cardiac-selective effects. While the degree and pattern of system effects differed across and within our analysis groups, it is apparent that any patient may be susceptible to a wide variety of symptoms. The Gly406Arg variant does appear most likely to cause a more severe phenotype with higher incidence of both cardiac and non-cardiac features, though some differences persist between TS1 and TS2 Gly406Arg, such as the presence of syndactyly which was statistically different across the groups (*p* < 0.0001), occurring primarily in TS1 individuals. Likewise, instances of bone and joint concerns differed across the groups (*p* = 0.0054), with higher incidence in TS1 Gly406Arg and TS2 Gly406Arg, and no occurrences in the Gly402Ser or diagnosed nsLQT8 cohorts. Also of note, Gly402Ser individuals differ from TS2 Gly406Arg in their lack of cardiac concerns *in utero* (*p* = 0.0345), leading to later recognition of the disease. Importantly, there was no statistical difference among groups for the presence of LQT or congenital heart defects, while the probability of arrythmia was higher in the TS1 group (*p* = 0.0168), but equivalent across the other 4 categories (*p* = 0.6638). Thus, every group, even those diagnosed with nsLQT8, demonstrated a potential for cardiac involvement. Moreover, neurological features were identified across all categories, with no difference in ASD or ADD/ADHD diagnosis across the different cohorts (*p* = 0.6873 and 0.2840 respectively). However, the presence of any neurodevelopmental concern (consisting of ASD, ADD, ADHD, global developmental delay or seizure) occurred with equal frequency across all groups except for those diagnosed with nsLQT8 (*p* = 0.0314), demonstrating the non-homogeneous pattern of features among patients. Overall, for most categories of symptoms, Gly402Ser, nsLQT8, and other *CACNA1C* variants were often difficult to distinguish, exhibiting overlapping symptoms that varied significantly across the patient population. Thus, we propose that Timothy syndrome and *CACNA1C* related disorder present as a spectrum of symptoms, with any patient harboring a *CACNA1C* allele at potential risk for both cardiac and non-cardiac phenotypes regardless of the initial diagnosis, making the nsLQT8 diagnosis a misnomer. At the same time, the severe disease first described by Splawski et. al. [10] represents the most severe manifestation of the disease, with many patients in this study reporting a less debilitating variation of the disease and significantly increased survival. In particular, the risk of sudden death was found to differ significantly across the groups (*p* = 0.0333), demonstrating a better prognosis for Gly402Ser and diagnosed nsLQT8 individuals. Finally, it is important to note that several of the respondents for children with other variants did not report any cardiac features. Given that most patients have historically been identified following a cardiac event, it is probable that patients without severe cardiac effects are underrepresented in this study. Yet, it is important to note that not all patients with reported LQT presented at birth, leaving open the possibility that patients initially diagnosed with normal or borderline QT intervals may be diagnosed later in life, and may be susceptible to acquired LQT.

### Life-threatening cardiac and extra-cardiac symptoms

Our study has revealed considerable new insights on three life-threatening conditions faced by TS individuals that were not apparent when the syndrome was first described. These include: (1) cardiac arrhythmias and SCA, (2) clinically significant hypoglycemia, and (3) defects in respiratory function.

#### Arrhythmia

Since the discovery of TS, LQTS and life-threatening cardiac arrhythmias have been the hallmark features. Individuals with TS are at risk of SCA due to underlying prolonged QT interval. Even those patients harboring a *CACNA1C* variant that does not present with LQT should be aware of potential cardiac risk. They also must avoid medications that further prolong QT interval to avoid risk of cardiac events [39]. All TS patients require prompt treatment; ICD implantation should be considered upon diagnosis to reduce the risk of mortality.

#### Hypoglycemia

Unusual dips in blood sugar are a demonstrated concern across all groups within this study (Table 4: Hypoglycemia and endocrine dysfunction), with no difference in the probability of occurrence across the different groups (*p* = 0.6123; Table 10: Cardiac medications and procedures). Hypoglycemia has been implicated as the underlying cause of death of at least one child with TS1. Although many hypoglycemic episodes appear to be associated with infection, some hypoglycemic episodes have no recognizable associations and appear to be spontaneous.

Hypoglycemia occurs commonly in TS newborns, particularly those born pre-term. In our study, 5/6 (83%) of hypoglycemic TS1 newborns were born pre-term. Whether this hypoglycemia is due to TS directly, or indirectly due to the metabolic stress of delivery in combination with insufficient glycogen reserves in pre-term newborns is unknown.

#### Respiratory dysfunction

Respiratory dysfunction in TS can be severe, manifesting as spontaneous respiratory arrest, and occurs comparably across all groups (*p* = 0.4695). One infant with TS2 Gly406Arg died following documented primary pulmonary hypertension without documented arrhythmia. Respiratory associated death was also noted in 3 additional patients, one with TS1 and 2 with other variants. Recurrent pneumonias in early childhood, especially in boys with TS1, followed by asthma requiring nebulizer use, are also noted to occur. The cause of respiratory dysfunction is unknown but could be associated with compromised pulmonary development consequent to premature birth and/or to abnormal lung function that occurs with Cesarean deliveries [40–42] which were prevalent obstetric complications in our study.

### Review of therapies and treatment options

#### Pharmacological therapy

As discussed above, most TS1 children and children with the Gly402Ser have received beta blockers for management of LQTS. Several reports suggest that calcium channel antagonists may also prove helpful. Among TS1 children in this study, one who could not tolerate beta blockers received verapamil. The therapeutic use of calcium channel blockers, such as dihydropyridines (DHPs) and verapamil, in TS has been an area of interest given their potential to attenuate the increased calcium influx caused by dysfunctional Ca_V_1.2 channels. These blockers inhibit L-type calcium channels, which could theoretically reduce the prolonged calcium currents and mitigate the arrhythmic risk in TS patients. Despite this potential, there is limited clinical experience with these agents as primary therapy in TS [18, 43, 44]. Anecdotal reports and small case series suggest mixed results. For instance, while verapamil has been used in some cases, its effectiveness has not been consistently demonstrated [43, 44]. This inconsistency may be due to the state-dependent effects of calcium channel antagonists, which renders them less competent in the context of the biophysical alterations associated with the TS variant [45]. However, a TS animal model reproducing the calcium overload and decreased sodium current mediated by calmodulin kinase, was created to mimic the electrophysiological phenotype on QT prolongation and reentrant arrhythmia susceptibility [46]. In this model, verapamil was shown to shorten the QT interval and uniform repolarization, leading to decreased reentry susceptibility. Further demonstration of this effect in patients with TS is awaited. Overall, more systematic research and clinical trials would be needed to evaluate the efficacy and safety of these channel blockers in the TS population.

#### ICDs, AEDs, and home monitoring

Decisions regarding ICD implantation in the TS population are complicated by young age at time of diagnosis. Most patients are too small for conventional transvenous systems or a subcutaneous device, resulting in the need for modified epicardial implants which are known to have a relatively high rate of complications and need for early reintervention. In our survey, many parents expressed a desire to avoid early ICD implant and rely instead on noninvasive monitoring (e.g. Owlet sock monitor during sleep or a pulse oximeter), with a standby AED always available for the first years few of life. Implantable loop recorders [47, 48] can be considered for arrhythmia surveillance, and for those with pacemakers, tachycardia detection algorithms can be carefully monitored, perhaps allowing early detection of non-sustained arrhythmias that may influence decisions regarding the timing of ICD implant, although neither of these modalities can provide an instantaneous alert should a sustained ventricular arrhythmia occur [49]. Close daily observation by parents and other knowledgeable caregivers (with an AED constantly nearby) remains critical for safe monitoring of TS patients without ICDs in place.

#### Left cardiac sympathetic denervation (LCSD)

As there are a limited number of TS children who have undergone LCSD surgery, the utility of this procedure on individuals with TS is still unclear. For TS1 children who underwent this procedure 3 of 7 have subsequently died (Table 10: Cardiac medications and procedures), though it is understood by ICD interrogation that 2/3 TS1 children died of non-arrhythmic causes (hypoglycemia and an extra-cardiac dysfunction). Unfortunately, it is challenging to make general recommendations for this procedure for any child with TS since the sample size is small and there have not been clinical trials with follow-up. Additionally, many parents express hesitancy about early surgical procedures, such as LCSD or ICD implantation, as anesthesia and infection can pose major risks.

#### Pre-term delivery

Our study raises questions regarding the necessity of early pre-term delivery for fetuses with TS and intermittent 2:1 AVB. Traditionally, there has been a tendency to consider pre-term delivery for TS fetuses with intermittent 2:1 AVB or bradycardia under the assumption that pre-term delivery may mitigate potential risks related to bradycardia [50]. However, whether the advantages of pre-term delivery outweigh those of continued *in utero* development is an open question. Future study of developmental outcomes in infants born without early recognized cardiac manifestations may shed light on the risks related to early delivery and potentially enhance the care and management of these patients.

#### Monitoring/treating hypoglycemia

Intermittent hypoglycemia has been associated with sudden death in TS children. Although this was reported in the original 2004 publication [10], our survey has revealed that greater awareness of this phenotype is necessary. It is clear from our surveys that parents monitor their children’s glucose levels and have developed strategies to prevent hypoglycemia after an overnight fast. Despite this, more studies are needed to understand the relationship between illness/infection and low blood glucose levels.

#### Awareness of anesthesia risks

Anesthesia remains a serious threat to TS individuals. Twenty-one of 30 (70%) TS1 and 8 of 11 (72%) TS2 Gly406Arg children have suffered serious arrythmias upon administration of anesthesia or during surgery for correction of syndactyly, hip alignment, or dental extractions. It remains questionable whether hypoglycemia resulting from pre-surgery fasting is linked to or exacerbates arrhythmias during anesthesia for children with TS.

#### Gastro-intestinal challenges

In over 30 years of following TS children, the esophageal-gastrointestinal concerns have been under recognized, assumed to be due to a lack of GI evaluations. However, our study indicates they are among the more pressing health concerns for immediate care and survival. Esophageal concerns in infants and young children, and the development of significant GI problems as they mature were identified across all analyzed groups. This emphasizes the need for further evaluation and clarification of care specifically for the concerning effects most commonly caused by constipation in young TS children, and the deterioration of renal function associated with constipation as they age. Given the known tissue expression of *CACNA1C* in smooth muscle (www.proteinatlas.org), we hope these findings promote greater interest in GI research in the TS context.

### Challenges, future diagnostic approaches, and future therapies

The various phenotypes and variable presentations of TS children has created diagnostic challenges since many of the features are present in non-TS syndromes or are common congenital abnormalities that are recognized independently of TS (e.g., syndactyly). Thus, the first recognition of TS may occur as a tragic SCA or hypoglycemia. Earlier recognition of the syndrome would provide life-saving opportunities. A first step is increased awareness among pediatricians and pediatric advanced practice providers through efforts such as this review. In this genomic century we are on cusp of broad whole genome sequencing. If this strategy becomes common practice and a component of prenatal screening, TS (and other genetic diseases) may become recognized at earlier stages and thus provide opportunities for intervention before life-threatening manifestations.

As a defined genomic disease, TS may be amenable to the growing armamentarium of gene-editing and other gene-based strategies. Recent progress offers a tantalizing example. Delivering antisense oligonucleotides to block expression of the exon containing the Gly406Arg variant in exon 8a promoted utilization of the alternative exon 8 and consequent expression of channels with normal inactivation kinetics in a rodent model expressing patient forebrain organoids [51]. Other strategies such as base editing may offer future opportunities.

## Conclusions

Overall, our study highlights both shared and unique symptoms among TS1, TS2 Gly406Arg, LQT8, and individuals with other *CACNA1C* variants. We find a variety of extra-cardiac symptoms, across all groups analyzed. Several of these features, such as gastrointestinal concerns and hypoglycemia, were identified in every group, suggesting that any variant in *CACNA1C* has the potential to be syndromic. We hope that our present study has brought to light the serious extracardiac phenotypes that can impact survival rate and quality of life. We believe a better understanding of the cardiac and extra cardiac concerns faced by these patients will lead to more comprehensive disease management and identify necessary avenues of TS research to improve treatments for patients.

## Electronic supplementary material

Below is the link to the electronic supplementary material.

Supplementary Material 1: Additional file 1. Timothy Syndrome Questionnaire. This file provides the questionnaire utilized in this study is provided as an additional file.

## Data Availability

Due to privacy concerns, the full datasets are not publicly available.

## Abbreviations

TS: Timothy syndrome
TS1: Timothy syndrome type 1
TS2: Timothy syndrome type 2
LQT: Long QT
LQT8: Long QT type 8
nsLQT8: Non-syndromic Long QT type 8
ADD: Attention deficit disorder
ADHD: Attention-deficit/hyperactivity disorder
ICD: Implantable cardiac defibrillator
AED: Automated external defibrillator
BB: Beta blocker
SUD: Sudden unexplained death
SADS: Sudden Arrythmia Death Syndromes
AVB: Atrioventricular block
PDA: Patent ductus arteriosus
PFO: Patent foramen ovale
VSD: Ventricular septal defect
HCM: Hypertrophic cardiomyopathy
ToF: Tetralogy of Fallot
CHD: Congenital heart defect
LVH: Left ventricular hypertrophy
SCA: Sudden cardiac arrest
LCSD: Left cardiac stellate denervation
