# Supplementary material for: A Natural History Study of Timothy Syndrome
Source: Orphanet J Rare Dis. 2024 Nov 23;19:433. doi: 10.1186/s13023-024-03445-x (PMC11585941; doi:10.1186/s13023-024-03445-x)
Supplement: Supplementary file 1 — Supplementary Material 1: Additional file 1. Timothy Syndrome Questionnaire. This file provides the questionnaire utilized in this study is provided as an additional file. [file 13023_2024_3445_MOESM1_ESM.pdf]

Timothy Syndrome Questionnaire

1. Email *

Please do not share the name of your child or the city in which you reside in any of your answers. Thank you for your participation in this survey.

Birth details

2. Birthdate

Example: January 7, 2019

3. Biological sex

4. Weeks gestation

5. Delivery

Mark only one oval.

- Vaginal birth
- Cesarean birth

6. Weight at birth (specify units)

7. Length at birth (specify units)

8. APGAR score at birth

9. Twins?

Mark only one oval.

- No
- Yes, identical
- Yes, fraternal

10. Did your child have any trouble breastfeeding?

11. Has your child's case ever been published in a medical journal? If so, please cite the article.

12. Please select any symptom that was noted at birth:

Check all that apply.

- [ ] Bradycardia
- [ ] 2:1 AVB
- [ ] Arrhythmia
- [ ] Long QT
- [ ] Hypoglycemia
- [ ] Hypotonia
- [ ] T-wave alternans
- [ ] Other: ______

13. If you selected other, please explain:

Heart details

14. Did your child have heart problems before birth? If yes, please explain.

15. If LQT was not noted at birth, when was it identified?

16. Were arrhythmias noted during the day or at night? During sleep or while awake?

17. Was a pacemaker placed at birth?

Mark only one oval.

- Yes
- No

18. If not, was a pacemaker placed some time after birth? At what age?

19. Was an ICD placed at birth?

Mark only one oval.

- Yes
- No

20. If not, was an ICD placed some time after birth? At what age?

21. Was a loop recorder ever placed? At what age?

22. Has your child ever had a fasting test and/or an exercise tolerance test? If so, please describe:

23. At what age was TS diagnosis made?

24. What variant in CACNA1C does your child have?

Mark only one oval.

- G406R Exon 8A
- G406R Exon 8
- G402S
- E407G
- S643F
- E1115K
- I1166T
- A1473G
- R1024G
- G1911R
- R518C
- R518H
- Other

25. If you selected other, what is your child's variant?

____

26. Were any additional genetic changes identified in the genetic results?

____

27. Were either of the parents genetically tested?

Mark only one oval.

- Yes
- No

28. If yes, what were the results?

____

Heart details

Please answer yes/no to whether or not your child has experienced any of the following cardiac abnormalities, and explain any "yes" answers.

29. Congenital heart defects (Tetralogy of Fallot, PDA, PFO, ASD, VSD, Coarc of aorta, valve defects, atresia, etc)

____

30. Hypertrophic cardiomyopathy (HCM)

31. Abnormal response to anesthesia (please list any noted arrhythmia)

32. Suffered cardiac arrest

33. Lead replacement problems

34. Received ICD shocks for arrhythmias

35. Inappropriate ICD shocks given

36. Arrhythmias noted at night

Triggers for cardiac episodes

37. Has your child ever experienced unusual weakness without cause? If yes, please explain:

38. Please select all stimuli you believe to be triggers for cardiac events:

Check all that apply.

- [ ] Loud noise
- [ ] Sleep
- [ ] Excitement
- [ ] Infections
- [ ] Fever
- [ ] Hypoglycemia
- [ ] Dehydration
- [ ] Other: ______________________

39. If you selected "other," please elaborate:

____
____
____
____
____

Medications

Please answer yes/no to whether your child is currently taking the following medications, and list the dosage for each:

40. Beta blockers (please note name)

____

41. Mexiletine

____

42. Amiodarone

____

43. Verapamil

____

Checkpoint 1

44. Do you want to save your answers and quit for now? You will be able to return to the survey at a later time.

Mark only one oval.

- Yes, save and quit
- No, continue

Extra-cardiac features

Bone/Skin/Muscle

45. Was your child born with syndactyly of the fingers? If yes, which fingers on which hand(s)?

____

46. Was your child born with syndactyly of toes? If yes, which toes on which foot?

____

47. Was either parent born with syndactyly?

____

48. Please select any of the following bone/skin symptoms your child has been diagnosed with:

Check all that apply.

- [ ] congenital hip dislocation, dysplasia, or other hip disorder
- [ ] club foot
- [ ] misplaced or abnormal thumb or finger placement
- [ ] foot inversion
- [ ] spinal cord syrinx
- [ ] ileal atresia
- [ ] hypotonia/low muscle tone
- [ ] other

49. If you selected other, please explain:

____
____
____
____
____

Extra-cardiac features

Craniofacial & skin-related

50. Please select any of the following craniofacial features a doctor has noted:

Check all that apply.

- [ ] Low set ears
- [ ] Flat nose bridge
- [ ] Small, depressed upper lip
- [ ] Round face
- [ ] Bald at birth

51. At what age did your child begin to grow hair?

____

52. Please select any of the following skin conditions a doctor has noted:

Check all that apply.

- [ ] Molting/excessive flaky skin
- [ ] Discoloration
- [ ] Psoriasis
- [ ] Sensitivity to hot/cold

53. If you selected any of the above conditions, what treatments (if any) have you used to manage symptoms?

____
____
____
____
____

54. Does your child prefer or get irritated by certain types of clothing? Please explain:

____
____
____
____
____

Extra-cardiac features

Dental

55. Does your child resist brushing teeth?

____

56. Please select any of the following dental symptoms your child has experienced:

Check all that apply.

- [ ] Frequent cavities
- [ ] Misplaced teeth
- [ ] Diseased gums
- [ ] Small teeth
- [ ] Underbite
- [ ] Overbite
- [ ] Diagnosed gum disease
- [ ] Dental surgeries/teeth extractions
- [ ] Successful use of braces
- [ ] Fluoride treatments

57. Please explain any of the symptoms you selected above, particularly diagnosed gum diseases or surgeries:

____
____
____
____
____

Extra-cardiac features

Eyes & ears

58. Does your child wear glasses? If yes, what is the prescription?

____

59. If you answered yes above, at what age were glasses prescribed?

____

60. Please select any of the following eye-related symptoms your child has experienced:

Check all that apply.

- [ ] Retina detachment
- [ ] Lazy eye
- [ ] Wandering eye
- [ ] Astigmatism
- [ ] Other

61. If you selected "other," please explain:

62. Did you child ever have an eye concern that required surgery? If so, please explain:

63. Does your child have any hearing loss? If so, please explain:

64. Does your child have frequent ear infections?

Mark only one oval.

- ( ) Yes
- ( ) No

65. Has your child had eustachian tubes placed?

66. Do sounds seem to irritate your child? If so, please explain:

Extra-cardiac features

Esophageal symptoms

67. Please select any of the following esophageal symptoms your child has experienced:

Check all that apply.

- [ ] Difficulty swallowing
- [ ] Frequent vomiting
- [ ] Aspiration of food or medications
- [ ] Gastric reflux
- [ ] Sensitivity to food textures
- [ ] Unusual or frequent gagging
- [ ] Other: ______________________

68. Please describe the details of any of the symptoms you selected above:

____
____
____
____
____

69. Medications taken for any of the above symptoms, and their dosages:

____
____
____
____
____

70. Has a feeding tube ever been placed? If yes, for how long?

____

71. Please select any of the following gastrointestinal symptoms your child has experienced:

Check all that apply.

- [ ] Frequent stomach aches
- [ ] Ulcers or other diagnosed stomach problems
- [ ] Diarrhea
- [ ] Constipation
- [ ] Food sensitivities or allergies

72. Medications taken for any of the above symptoms, and their dosages:

73. Is there a seasonal nature to these symptoms? If yes, please explain:

Checkpoint 2

74. Do you want to save your answers and quit for now? You will be able to return to the survey at a later time.

Mark only one oval.

- Yes, save and quit
- No, continue

Extra-cardiac features

Laryngeal, respiratory, & immune

75. Did your child learn and respond to sign language before learning to speak?

76. At what age did your child begin to speak?

77. At what age did your child begin to speak in full sentences?

78. Did your child ever see a Speech Language Therapist?

79. Please select any of the following laryngeal symptoms your child has experienced:

Check all that apply.

- [ ] Expressive language delay
- [ ] Produced odd sounds
- [ ] Voice impairment
- [ ] Lisps or stuttering
- [ ] Other diagnosed speech defect

80. If you selected other, please explain:

____
____
____
____
____

81. If you selected any of the above symptoms, at what age did they arise?

____

82. If you selected any of the above symptoms, have they resolved? What interventions were effective?

____
____
____
____
____

83. Please select any of the following respiratory symptoms your child has experienced:

Check all that apply.

- [ ] Frequent pneumonia
- [ ] Laryngitis
- [ ] Bronchitis
- [ ] Asthma

84. Medications taken for any of the above symptoms, and their dosages:

____
____
____
____
____

85. Does your child experience frequent infections or other immune problems? If yes, please explain:

86. Has your child ever had their immune system tested? If yes, please explain:

87. Please list any medications or treatments for immune problems:

88. Is there a seasonal nature to these symptoms? If yes, please explain:

89. Is illness ever associated with hypoglycemia? Please explain:

90. Have you noticed a delay in wound healing? Please explain:

91. Does your child have allergies or hay fever? If yes, please specify:

92. If you answered yes, does your child take any medications for allergies or hay fever?

93. Has your child ever been treated for sepsis?

Extra-cardiac features

Endocrine

94. Is your child currently average size, overweight, or underweight?

Mark only one oval.

- ( ) Average
- ( ) Overweight
- ( ) Underweight

95. Please comment on any endocrine medications and their dosages:

96. Has your child ever experienced hypokalemia, hyperkalemia, and/or hypercalcemia?

97. Has your child experienced early-onset puberty?

Mark only one oval.

- Yes
- No
- N/A

98. If female, at what age did your daughter first menstruate? Does she now have regular monthly menstruation?

____

99. Does your child experience frequent sleep disturbances?

Mark only one oval.

- Yes
- No

100. If yes, do you use any sleep aids such as melatonin? Please specify:

____

101. Has your child ever been diagnosed with thyroid problems?

____

102. Has your child ever been diagnosed with abnormal cortisol levels?

____

103. Have any other hormone abnormalities been diagnosed for your child? If yes, please specify:

____

Extra-cardiac features

Hypoglycemia

104. Has your child ever experienced hypoglycemia?

Mark only one oval.

- Yes
- No *Skip to question 109*

Extra-cardiac features

Hypoglycemia

105. How many episodes of hypoglycemia have you noted?

106. What was the measured blood sugar level?

107. What were the symptoms of hypoglycemia that you noted?

108. What protocols, if any, do you use to stabilize blood sugar during sleep?

Skip to question 110

Extra-cardiac features

Hypoglycemia

109. What protocols, if any, do you use to manage blood sugar?

Checkpoint 3

110. Do you want to save your answers and quit for now? You will be able to return to the survey at a later time.

Mark only one oval.

- ( ) Yes, save and quit
- ( ) No, continue

Extra-cardiac features

Excretory System

111. Has your child ever had a kidney stone?

112. Has your child ever experienced renal reflux? If so, what medications do they take for this condition?

113. Has your child ever had a urinary tract infection (UTI)?

114. If yes, how frequently?

115. Has your child ever had a diagnosis of structural kidney or urinary abnormalities? If so, what is the diagnosis?

116. At what age was your child potty trained?

117. Has your child ever had trouble with bladder control at night? If so, at what age did this stop?

118. Has your child ever been diagnosed with kidney disease? If so, what is the diagnosis and what is the current stage of disease?

Extra-cardiac features

Brain development

For each of the following, please note the age at which your child developed these skills:

119. Holding head up

120. Sitting

121. Crawling

122. Standing alone

123. Walking

124. Running

125. Jumping

126. Skipping

127. Climbing

128. Riding a bike

129. Has your child ever been a "toe walker?"

130. Has your child ever had uncontrolled or involuntary tics?

____

131. Has your child ever had uncontrolled or involuntary noise-making (barking, burping, humming, throat clearing, etc)?

____

132. Has your child ever exhibited strange or uncontrolled body movements or contortions?

____

133. Has your child ever had a brain scan/MRI?

____

134. Has your child ever been diagnosed with epilepsy or a seizure disorder?

____

Extra-cardiac features social

Social & emotional development

135. Please select any of the following features that describe your child:

Check all that apply.

- [ ] Overly friendly
- [ ] Overly anxious or shy
- [ ] Odd behaviors
- [ ] Plays easily with age group
- [ ] Prefers being alone
- [ ] Prefers quiet environment
- [ ] Prefers animals over people
- [ ] Prefers specific ordering of objects
- [ ] Routine-oriented
- [ ] Echolalia

136. Does your child seek sensory input in atypical ways, i.e. banging head, pinching/biting self?

____

137. Please select any of the following disorders with which your child has been diagnosed:

Check all that apply.

- [ ] Phobias
- [ ] Over-reactive anger or violence
- [ ] Depression
- [ ] ADD/ADHD
- [ ] Schizophrenia
- [ ] Anxiety
- [ ] OCD
- [ ] Autism
- [ ] Chronic headaches or migraines
- [ ] Other diagnosis

138. If you selected other, please explain:

139. Medications taken for any of the above symptoms, and their dosages:

140. Is there a seasonal nature to these symptoms? If yes, please explain:

Innoculations

141. Did your child receive vaccinations at the recommended ages?

142. Did your child experience any unusual health or behavioral changes with specific vaccinations? If so, please specify which vaccine and the changes.

Education

143. At what age was your child able to name the letters in the alphabet according to your native language?

144. Does your child perform at grade level? If not, what tutoring or accommodations has your child been given?

145. Please select any of the following educational features that describe your child:

Check all that apply.

- [ ] Problems with reading
- [ ] Problems with numbers
- [ ] Unusually strong ability to remember rote facts
- [ ] Other specific giftedness or talents

146. Please elaborate on any features you selected:

Miscellaneous features

147. At what age did your child begin to sleep through the night?

148. Please select any of the following features that describe your child:

Check all that apply.

- [ ] Frequent fluctuations in body temperature
- [ ] Blood pressure problems
- [ ] Frequent nightmares
- [ ] Unusually small veins
- [ ] Unusual swelling
- [ ] Coordination issues
- [ ] Other

149. If you selected any of the above features, please explain.

150. Medications taken for any of the above symptoms, and their dosages:

Final questions

151. Are there any other features you may have noticed that were not mentioned in this survey?

152. Does your child take any other medications or supplements that you have not yet listed in this survey? Please indicate the dosages and for what conditions.
